# Ergothioneine Biosynthesis and Functionality in the Opportunistic Fungal Pathogen, *Aspergillus fumigatus*

**DOI:** 10.1038/srep35306

**Published:** 2016-10-17

**Authors:** Kevin J. Sheridan, Beatrix Elisabeth Lechner, Grainne O’ Keeffe, Markus A. Keller, Ernst R. Werner, Herbert Lindner, Gary W. Jones, Hubertus Haas, Sean Doyle

**Affiliations:** 1 Department of Biology, Maynooth University, Maynooth, Co. Kildare, Ireland; 2 Division of Molecular Biology, Biocenter, Medical University Innsbruck, Innrain 80/82, Austria; 3 Division of Biological Chemistry, Biocenter, Medical University Innsbruck, Innrain 80/82, Austria; 4 Division of Clinical Biochemistry, Biocenter, Medical University Innsbruck, Innrain 80/82, Austria

## Abstract

Ergothioneine (EGT; 2-mercaptohistidine trimethylbetaine) is a trimethylated and sulphurised histidine derivative which exhibits antioxidant properties. Here we report that deletion of *Aspergillus fumigatus egtA* (AFUA_2G15650), which encodes a trimodular enzyme, abrogated EGT biosynthesis in this opportunistic pathogen. EGT biosynthetic deficiency in *A. fumigatus* significantly reduced resistance to elevated H_2_O_2_ and menadione, respectively, impaired gliotoxin production and resulted in attenuated conidiation. Quantitative proteomic analysis revealed substantial proteomic remodelling in Δ*egtA* compared to wild-type under both basal and ROS conditions, whereby the abundance of 290 proteins was altered. Specifically, the reciprocal differential abundance of cystathionine γ-synthase and β-lyase, respectively, influenced cystathionine availability to effect EGT biosynthesis. A combined deficiency in EGT biosynthesis and the oxidative stress response regulator Yap1, which led to extreme oxidative stress susceptibility, decreased resistance to heavy metals and production of the extracellular siderophore triacetylfusarinine C and increased accumulation of the intracellular siderophore ferricrocin. EGT dissipated H_2_O_2_ *in vitro*, and elevated intracellular GSH levels accompanied abrogation of EGT biosynthesis. EGT deficiency only decreased resistance to high H_2_O_2_ levels which suggests functionality as an auxiliary antioxidant, required for growth at elevated oxidative stress conditions. Combined, these data reveal new interactions between cellular redox homeostasis, secondary metabolism and metal ion homeostasis.

## Introduction

**Figure 1:**
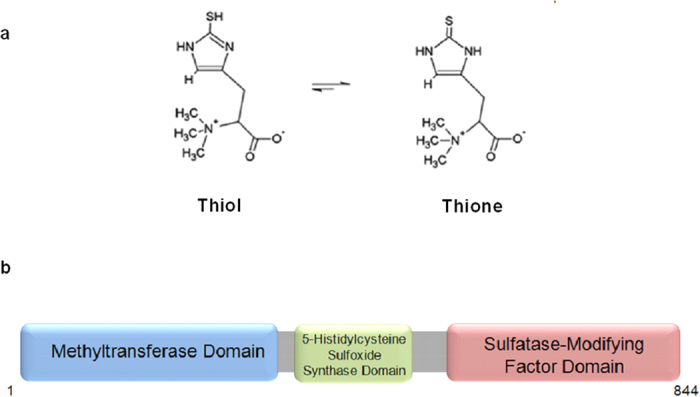
Ergothioneine displayed in both thiol and thione form and EgtA structure. (**a**) The structure of ergothioneine in both thiol and thione form. (**b**) EgtA domain architecture, 844 amino acids, domains include a methyltransferase domain at the N-terminal end, a sulfatase-modifying factor enzyme 1 at the C-terminal and an internal 5′-histidylcysteine sulfoxide synthase domain.

Ergothioneine (2-mercaptohistidine trimethylbetaine; EGT) is derived from histidine and exists in a tautomeric state between both the thione and thiol forms (Fig. 1a)^1^. EGT exhibits a high redox potential (−0.06 V) and so is classified as a powerful antioxidant^2^,^3^. Humans cannot biosynthesize EGT and acquire it in the diet from both plant and animals sources, however it appears that EGT biosynthesis only occurs in specific bacterial and fungal species^4^. Human cells possess a receptor, termed OCTN1, which facilitates EGT uptake, and this receptor is highly abundant in trachea, ileum and kidney cells as well as CD71^+^ cells from bone marrow, cord blood and fetal liver^5^. Although its dietary antioxidant properties have been extensively studied^6^,^7^,^8^,^9^,^10^,^11^, it remains to be conclusively proven that EGT has demonstrable health benefits in humans.

The seminal work of Seebeck^12^ revealed the mechanism of EGT biosynthesis in *Mycobacterium smegmatis*, which involves five discrete enzymes (EgtA-E). It is now clear that EGT biosynthesis in bacteria requires trimethylation of the NH_2_ group of histidine to generate hercynine. γ-glutamylcysteine (a thiol source) is then conjugated to the imidazole side chain of hercynine, followed by subsequent enzymatic processing to yield EGT. The crystal structures of EgtB, EgtC and EgtD have been recently determined^13^,^14^,^15^. In filamentous fungi, *egt-1* has been demonstrated to be essential for EGT biosynthesis in *Neurospora crassa*^16^. This gene encodes a protein with both *S*-adenosyl methionine (SAM)-dependant methyltransferase and formylglycine-generating enzyme (FGE) sulphatase domains. Egt-1 is postulated to carry out the first two steps of EGT biosynthesis: trimethylation of histidine to hercynine, followed by sulphoxidation of the hercynine to hercynylcysteine sulphoxide. The final step in EGT biosynthesis converts hercynylcysteine sulphoxide to EGT via a pyridoxal phosphate requiring enzyme, possibly encoded independently of *egt-1*. Analysis of an *Ncegt-1* deletion strain revealed increased conidial sensitivity to peroxide, but not superoxide or Cu^2+^, during germination. EGT also appeared to protect conidia in the time-period between conidiogenesis and germination. No other significant phenotypes associated with absence of EGT were observed in the *Ncegt-1* deletion strain^16^.

In *Schizosaccharomyces pombe*, EGT biosynthesis has been postulated to require only two genes, namely *egt1*, which showed domain and functional homology to EgtB and EgtD of *M. smegmatis* and *egt2* as a possible homolog of *egtE* from *M. smegmatis*^17^. Because fungi appear to lack the enzyme which removes the glutamyl residue from a biosynthetic intermediate in bacterial EGT biosynthetic process^12^, *N. crassa* and *S. pombe* use cysteine rather than γ–glutamylcysteine to effect EGT formation. Deficiency of *S. pombe egt1* results in the loss of EGT production. If *egt2* is missing, minor amounts of EGT can be found possibly due to the spontaneous reaction of hercynylcysteine sulphoxide catalyzed by an unrelated pyridoxal phosphate (PLP)-binding enzyme. Interestingly, Pluskal *et al.*^17^ found no impact of EGT on the resistance against oxidative stress and argued that the antioxidant might not play a role in primary defense as loss is compensated by other mechanisms. Thus, details of the precise role of EGT in fungi are outstanding.

Although EGT presence has been studied extensively in *Basidiomycetes* as a source of dietary antioxidants, minimal data exist relative to the presence or function of EGT in pathogenic fungi, especially *Aspergillus fumigatus*. Relevantly, Gallagher *et al.*^18^ were the first to detect EGT in *A. fumigatus*, a fungal airborne pathogen which causes severe allergic or invasive diseases in immunosuppressed individuals. Gallagher *et al.*^18^ revealed that disruption of gliotoxin biosynthesis at a specific step (deletion of the γ-glutamyl cyclotransferase *gliK*), concomitantly resulted in significant oxidative stress and significantly elevated EGT levels in *A. fumigatus*. Notably, apart from that work representing the first identification of EGT in *A. fumigatus*, it presented a novel alkylation strategy, utilizing 5′-iodoacetamidofluorescein (5′-IAF), combined with either reverse-phase high-performance liquid chromatography (RP-HPLC) or LC-mass spectrometry (LC-MS), to detect EGT. An identical strategy has subsequently been deployed by others, using capilliary electrophoresis, to determine EGT levels in human plasma^19^,^20^.

Oxidative stress can also arise from altered iron metabolism in *A. fumigatus*. Iron is an essential trace element and is involved in various cellular processes. In excess, iron can produce oxidative stress via the Haber-Weiss/Fenton chemistry^21^. In *A. fumigatus*, iron limitation leads to the formation of low molecular mass ferric iron-specific chelators (siderophores), which are either excreted as extracellular siderophores, like fusarinine C (FsC) or triacetylfusarinine C (TAFC) to import Fe^3+^ into the fungus, or intracellular siderophores ferricrocin (FC) or hydroxyl-ferricrocin (HFC) to store or transport iron to other parts of the hyphae or conidia^22^,^23^,^24^,^25^,^26^. Lack of siderophores and subsequent lower uptake of iron led to attenuated virulence in *A. fumigatus*, which was shown in neutropenic mice^25^. Furthermore, the inability to protect against oxidative stress by deficiency in protein phosphatase Z, PhzA, leads to defective virulence of *A. fumigatus* in the immunocompetent murine model of corneal infection^27^. A key component of oxidative stress defense in *A. fumigatus* is Yap1. This transcriptional regulator has been shown to play an important role in coordinating the oxidative stress response and its deletion in *A. fumigatus* results in sensitivity to damage via elevated ROS levels^28^.

Here we describe the identification of a key EGT biosynthetic gene in *A. fumigatus* and reveal new insights into systems biology of EGT biosynthesis and functionality in fungi.

## Results

### Characterization and bioinformatic analysis of EgtA in *A. fumigatus*

The biosynthesis of EGT was first described in the prokaryote *M. smegmatis* and recent genetic comparative studies and homology searches identified the biosynthetic genes in *N. crassa* and *S. pombe*^16^,^17^. Blast searches (http://blast.ncbi.nlm.nih.gov/Blast.cgi) revealed possible homologous enzymes to *M. smegmatis* EgtB and EgtD, which are involved in the EGT biosynthesis annotated as DUF323 (AFUA_2G15650; *egtA*) in *A. fumigatus*, NCU04343 (*NcEgt-1*) in *N. crassa*^16^, and SPBC1604.01 (*egt1*) in *S. pombe*^17^. Alignments and genome-wide in-depth phylogenetic analyses showed that a gene fusion occurred possibly during the evolution in bacterial species^4^ and that EgtA from *A. fumigatus* also exhibited the fusion product of the two enzymes of *M. smegmatis* as seen in the aforementioned fungal species. In *A. fumigatus*, EgtA comprises 844 amino acids, has domains of a histidine-specific SAM-dependent methyltransferase at the N-terminal end, a sulphatase-modifying factor enzyme 1 at the C-terminal end and an intervening 5-histidylcysteine sulphoxide synthase domain (Fig. 1b). The 5′UTR comprises 902 nucleotides, the 3′UTR extends 265 nucleotides and the coding sequence is interrupted by six introns, whereby the genomic DNA is 2890 nucleotides long (http://www.aspergillusgenome.org/cgi-bin/locus.pl?locus=AFUA_2g15650&organism=A_fumigatus_Af293).

### *egtA* deletion and complementation

*A. fumigatus egtA* was deleted from strains AfS77 (a Δ*akuA::loxP* strain derived from ATCC46645 lacking non-homologous recombination^29^) and ATCC26933 using a split marker strategy^29^. Both strains were deployed as they produce low and high amounts of gliotoxin, respectively, and the impact of *egtA* deletion on gliotoxin biosynthesis was of relevance, since intracellular EGT levels significantly increase in a gliotoxin-deficient strain of *A. fumigatus*^18^,^30^. Deletion of *egtA* was confirmed via Southern analysis (Supplementary Figures S1 and S2) and abolition of gene expression was confirmed via RT-PCR (Supplementary Figure S3). Complementation of *egtA* in ∆*egtA*^26933^ was achieved using an alternative resistance marker as confirmed by Southern analysis (Supplementary Figure S4), and *egtA* expression was restored in *A. fumigatus ∆egtA*^26933^, as confirmed via RT-PCR (Supplementary Figure S3). *egtA* deletion was also successfully achieved in *A. fumigatus ∆yap1*^AfS77^, to yield *∆egtA*∆*yap1* and complementation of *egtA* in this double mutant was demonstrated (Supplementary Figure S2).

### Absence of EGT biosynthesis in *A. fumigatus* Δ*egtA*

**Figure 2:**
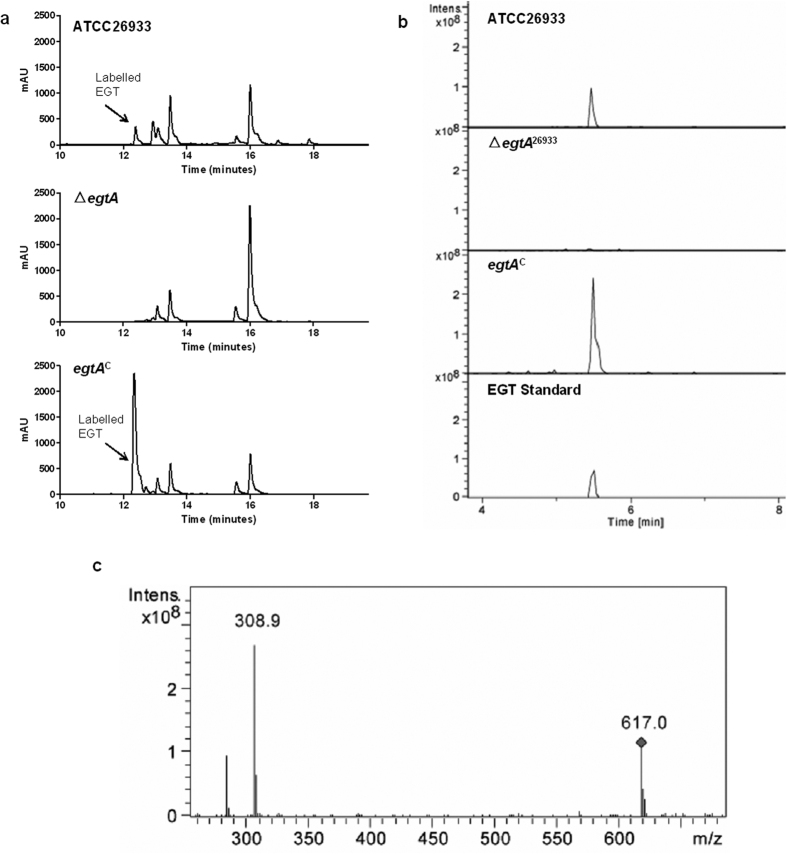
EGT detection via RP-HPLC and LC-MS in ATCC26933, Δ*egtA*^26933^ and *egtA*^C26933^. (**a**) RP-HPLC Chromatograms showing the detection of 5′-IAF labelled EGT. EGT was detected at a retention time of 12.4 min. EGT was detected in the wild-type and complemented sample at 12.4 min, but is absent from Δ*egtA.* (**b**) Extracted Ion Chromatographs (m/z: 617) following LC-MS analysis of TCA precipitated 5′-IAF-labelled protein extracts from ATCC26933, Δ*egtA*^26933^ and *egtA*^C26933^, in addition to an EGT standard. A peak at 5.3 min was confirmed to be EGT. This peak was absent from the Δ*egtA*^26933^ fraction, confirming the absence of EGT from the mutant. (**c**) Signature Ion breakdown corresponding to 5′-IAF labelled EGT.

Analysis of EGT biosynthesis was undertaken via RP-HPLC and LC-MS. 5′-IAF-alkylated mycelial lysates of wild-type, Δ*egtA*^26933^ and *egtA*^C26933^ were compared to commercially available EGT and revealed alkylated EGT in the wild-type and complemented strains at a retention time of 12.4 min. EGT was absent in Δ*egtA* mycelial lysates (Fig. 2a). EGT levels in *egtA*^C26933^ were elevated compared to wild-type. LC-MS analysis of TCA-precipitated 5′-IAF labelled mycelial lysates further confirmed the presence of EGT in the wild-type and complemented strains and its absence from *A. fumigatus* Δ*egtA* (Fig. 2b). Consistent with the ATCC26933, *egtA* deletion blocked EGT production also in *A. fumigatus* strain AfS77 strain (data not shown).

### H_2_O_2_ and menadione significantly impair growth of Δ*egtA* and exacerbate Δ*yap1* phenotype in Δ*egtA*Δ*yap1* during oxidative and heavy metal stress

**Figure 3:**
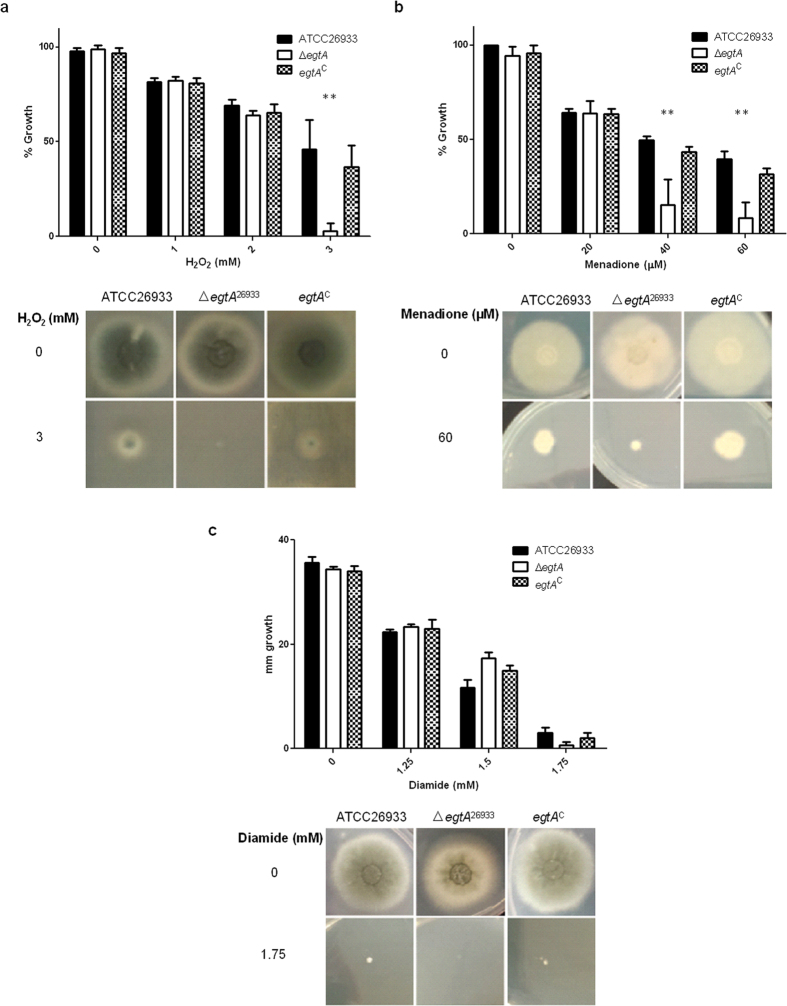
Plate assays performed on AMM agar (containing 5 mM ammonium tartrate as nitrogen source) for 72 h to test for sensitivity to various ROS inducing agents. (**a**) Plate assay with H_2_O_2_ ranging from 0 to 3 mM. Δ*egtA*^26933^ shows a significant (P = 0.0081) reduction in growth compared to ATCC26933 and *egtA*^C^ at 3 mM H_2_O_2_. (**b**) Plate assay with menadione ranging from 0 to 60 μM. Δ*egtA*^26933^ shows significantly reduced growth compared to ATCC26933 and *egtA*^C^ at both 40 μM (p = 0.0043) and 60 μM (P = 0.0013) menadione. (**c**) Plate assays with diamide ranging from 1.25 to 1.75 mM. Δ*egtA*^26933^ shows no significant difference in growth compared to ATCC26933 or *egtA*^C^.

**Figure 4:**
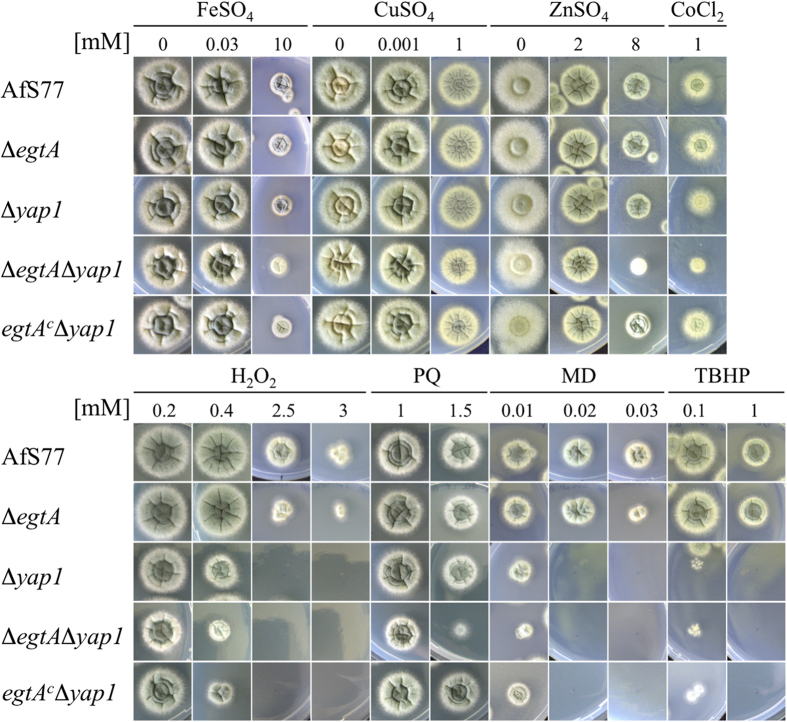
Plate assays performed on AMM agar (containing 20 mM L-glutamine as nitrogen source) for 48 h to test for sensitivity to various ROS inducing agents and heavy metals. Concentrations of stress-inducing agents (hydrogen peroxide, H_2_O_2_; paraquat, PQ; menadione, MD; *tert-*butylhydroperoxide, TBHP) and metals (iron, FeSO_4_; copper, CuSO_4_; zinc, ZnSO_4_; cobalt, CoCl_2_) are given in mM; All strains are AfS77 derivatives and 10^4^ spores were spotted. In wild-type-background EGT deficiency (strain ∆*egtA*) impaired resistance against H_2_O_2_ and MD and in a ∆*yap1*-background (strain ∆*egtA*∆*yap1;* Yap1 is a transcriptional activator orchestrating oxidative stress defense^28^) additionally against PQ and the metals copper, zinc and cobalt and iron. Complementation of EGT deficiency in the double mutant strain (*egtA*^*c*^∆*yap1*) restored phenotype to that of a Yap1-lacking mutant.

Plate assays to assess H_2_O_2,_ menadione and diamide sensitivity in ATCC26933, Δ*egtA*^26933^ and *egtA*^C26933^ revealed that Δ*egtA*^26933^ displayed increased sensitivity to H_2_O_2_ and menadione, but not diamide. After 72 h growth on *Aspergillus* minimal media (AMM) agar containing 3 mM H_2_O_2_, radial growth of Δ*egtA*^26933^ was significantly (P = 0.0081) reduced compared to wild-type and complemented strains. At 1 mM and 2 mM H_2_O_2_ however, Δ*egtA*^26933^ radial growth was unaffected compared to the wild-type (Fig. 3a). Wild-type growth was abolished at 4 mM H_2_O_2_, indicating EGT loss only effects protection from H_2_O_2_ at near lethal doses in *A. fumigatus* and positions it as an ‘antioxidant of last resort’. A significant decrease in radial growth was seen in Δ*egtA*^26933^ when exposed to menadione, at 40 μM (P = 0.0043) and 60 μM (P = 0.0013) (Fig. 3b). No sensitivity to diamide was observed within a range of 1.25 mM–1.75 mM (Fig. 3c). To further analyze the function of EGT against oxidative burden and metal toxicity, ∆*egtA*^AfS77^ (10^4^ spores/dot) were spotted on AMM agar containing the indicated amount of stress-inducing agents or metals, which was incubated up to 48 h at 37 °C (Fig. 4). EGT deficiency in the AfS77 background reduced resistance to hydrogen peroxide (3 mM H_2_O_2_) and menadione (20 μM) in ∆*egtA*^AfS77^. Moreover, the redox sensitivity phenotype of *A. fumigatus* ∆*yap1* was further exacerbated if EGT was absent (∆*egtA*∆*yap1*) whereby susceptibility was increased upon H_2_O_2_ (0.4 mM), paraquat (1.5 mM PQ), menadione (10 μM MD), *tert*-butylhydroperoxide (TBHP), iron, zinc, copper and cobalt exposure, respectively. EGT limitation also reduced sporulation of ∆*egtA*∆*yap1* at low zinc and at high concentrations of zinc, iron and copper (Fig. 4). The complemented strain *egtA*^c^∆*yap1* showed the same phenotype as the ∆*yap1* strain.

### Comparative Label Free Quantitative (LFQ) Proteomics Reveals Dysregulation of Redox-related Proteins in Δ*egtA* ^26933^ in response to H_2_O_2_

LFQ proteomic analysis revealed significant differences between ATCC26933 and Δ*egtA*^26933^. A comparison of Δ*egtA*^26933^ and ATCC26933 under basal conditions showed that absence of EGT biosynthesis resulted in a major proteomic adjustment in *A. fumigatus*. Specifically, the abundance of 26 proteins was increased or unique in Δ*egtA*^26933^ compared to wild-type, while 121 proteins were decreased in abundance or absent (Supplementary Table S1). Comparison of the proteomic profiles of Δ*egtA*^26933^ and ATCC26933 under H_2_O_2_-induced stress revealed an exacerbation of the differences between the mutant and wild-type. When exposed to 3 mM H_2_O_2_, the abundance of 290 proteins was dysregulated in the mutant compared to the wild-type, with 250 proteins increased or unique in Δ*egtA*^26933^ compared to ATCC26933, while 40 underwent reduced abundance or were absent (Supplementary Table S2). Many proteins which underwent dysregulated abundance under both basal and ROS conditions were reductases, oxidases, stress response proteins and enzymes with oxidising products (Supplementary Tables S3 and S4). This strongly suggests a disruption of redox homeostasis and oxidative stress defence in the Δ*egtA*^26933^, compared to ATCC26933, particularly when exposed to 3 mM H_2_O_2_. Of particular interest is redox sensitive subunit of the CCAAT-binding complex (CBC), HapC. Previously, the CCAAT-binding complex has been shown to be involved in oxidative stress response^31^. Therefore the absence of HapC in Δ*egtA*^26933^ compared to wild-type under basal conditions could be indicative of a defective oxidative defence system.

**Figure 5:**
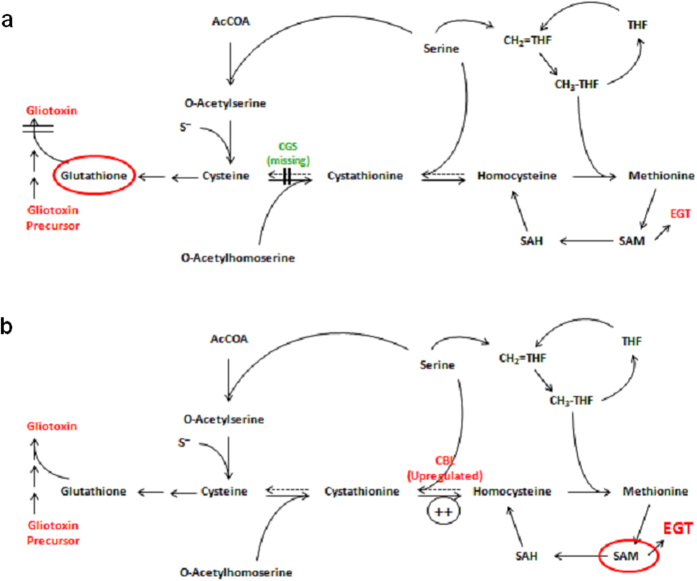
Metabolic pathways linking cystathionine, glutathione and methionine metabolism. (**a**) Comparison of Δ*egtA*^26933^ and ATCC26933 under basal conditions showing an absence of cystathionine γ-synthase, which converts cysteine to cystathionine. This could be due to a switch towards increased glutathione production. (**b**) Comparison of Δ*egtA*^26933^ and ATCC26933 upon addition of 3 mM H_2_O_2_ shows an increased abundance of cystathionine β-synthase, which converts cystathionine to homocysteine. This would result in increased production of SAM, which is required for EGT biosynthesis.

Cystathionine is a sulphur-containing metabolite involved in cysteine and methionine metabolism (Fig. 5), and the abundance of two proteins with activities related to cystathionine metabolism was dysregulated when the proteomic profiles of Δ*egtA*^26933^ and ATCC26933 were compared under both basal and H_2_O_2_-stressed conditions. Under basal conditions, cystathionine γ-synthase (CGS; AFUA_7G01590) was found to be absent in Δ*egtA*^26933^ compared to the wild-type. CGS catalyzes the formation of cystathionine from homoserine and cysteine^32^ and its absence in Δ*egtA*^26933^ suggests that cystathionine production is attenuated when EGT biosynthesis cannot occur, perhaps to provide cysteine in order to increase GSH production. Comparing Δ*egtA*^26933^ and ATCC26933 upon addition of H_2_O_2_, there was a significant increase (log_2_ 3.1-fold) in cystathionine β-lyase (CBL; AFUA_4G03950). CBL catalyses the conversion of cystathionine to homocysteine, ammonia and pyruvate^32^. Homocysteine can be converted into methionine, required for SAM biosynthesis. EGT biosynthesis requires SAM for the tri-methylation step via EgtA. Thus, a shift towards increased homocysteine formation, via CBL, in response to H_2_O_2_ addition could be part of an overall transition towards increasing SAM availability for EGT biosynthesis.

RT-qPCR analysis of CGS and CBL gene expression revealed a similar pattern to proteomic abundance alteration (Supplementary Figure S5). The CGS gene showed a non-significant reduction in expression in Δ*egtA*^26933^ compared to wild-type under basal conditions. A significant (P = 0.003) increase in the CBL gene was seen in Δ*egtA*^26933^ compared to wild-type under ROS conditions.

It was noted that no significant change in abundance of these proteins (CGS and CBL) was observed when comparing Δ*egtA*^26933^ under basal conditions to Δ*egtA*^26933^ under ROS conditions (Supplementary Table S5). This suggests that these changes are only significant when compared to the ATCC26933 response when exposed to 3 mM H_2_O_2_. This further illustrates the proteomic remodeling *A. fumigatus* undergoes when EGT biosynthesis is impeded and the mutant is forced to deal with oxidative stress.

H_2_O_2_ addition to Δ*egtA*^26933^ resulted in increased abundance of imidazole glycerol phosphate synthase subunit HisF (AFUA_ 2G06230) of log_2_ 1.11-fold compared to ATCC26933 under the same conditions. HisF is involved in histidine biosynthesis catalyzing the closure of the imidazole ring^33^. The observation of an increased abundance of imidazole glycerol phosphate synthase subunit HisF in Δ*egtA*^26933^ compared to wild-type under oxidative stress suggests that histidine production is increased when *A. fumigatus* is exposed to H_2_O_2_ in the absence of EGT biosynthesis. This could further indicate a shift towards attempting to synthesize EGT in the mutant, given that histidine is a key biosynthetic precursor of EGT.

A log_2_ 2.22-fold increase in a putative sulphite reductase (AFUA_2G15590; Supplementary Table S2) in Δ*egtA*^26933^ under oxidative stress compared to wild-type indicates an increased need for sulphur in Δ*egtA*^26933^, which could reflect attempted EGT production or an increase in GSH biosynthesis.

### *egtA* expression, but not EGT levels, increases in response to H_2_O_2_ exposure

**Figure 6:**
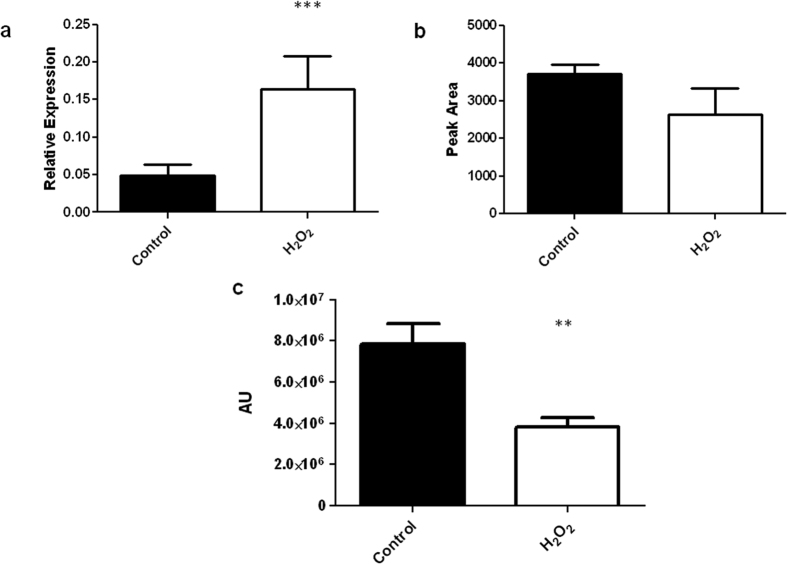
e*gtA* expression and EGT detection following 3 mM H_2_O_2_ exposure. (**a**) Quantitative RT-PCR data showing the relative expression of *egtA* in ATCC26933 following exposure to 3 mM H_2_O_2_. *egtA* levels are significantly (P = 0.0002) increased in ATCC26933 when exposed to H_2_O_2_ compared to control levels. (**b**) RP-HPLC analysis of 5′-IAF labelled EGT following ATCC26933 exposure to 3 mM H_2_O_2_. No significant variation in EGT levels was observed in H_2_O_2_ samples compared to control. (**c**) RP-HPLC data showing peak area of 5′-IAF labelled EGT following purified EGT incubation with 3 mM in H_2_O_2_. EGT levels drop significantly (P = 0.029) following 3 h reaction with 3 mM H_2_O_2_.

Quantitative RT-PCR of ATCC26933 cultures exposed to 3 mM H_2_O_2_ for 1 h in Sabouraud Dextrose broth showed a significant (P = 0.0002) increase in *egtA* expression in response to H_2_O_2_ compared to the control (Fig. 6a). This indicates H_2_O_2_ induces *egtA* expression. Unexpectedly, no corresponding increase in EGT levels was observed in ATCC26933 mycelial lysates following H_2_O_2_ exposure, which suggested that EGT may react to dissipate H_2_O_2_ (Fig. 6b). It was observed that treatment of purified EGT with H_2_O_2_ caused a significant decrease (~50%; P = 0.0029) in levels of the antioxidant (Fig. 6c), which is in accordance with EGT consumption by reaction with H_2_O_2_, and infers that *egtA* expression is increased to maintain intracellular EGT homeostasis.

### GSH levels in Δ*egtA* ^26933^ are increased compared to wild-type and complement

**Figure 7:**
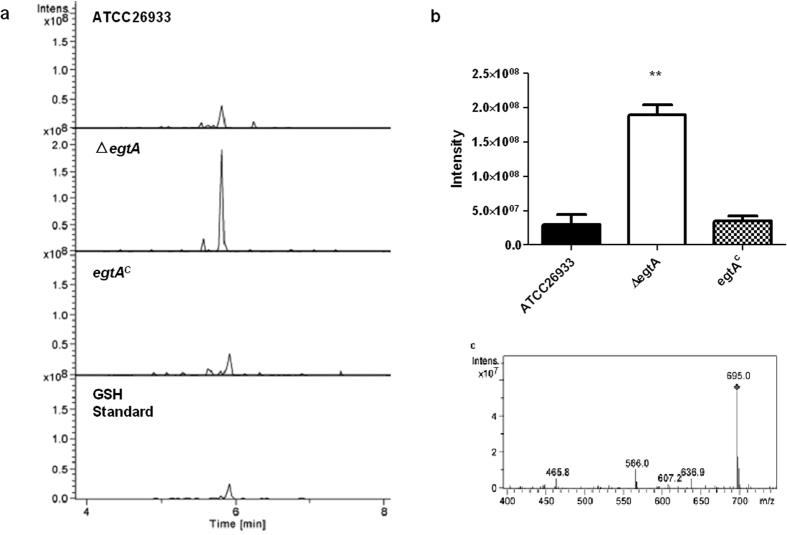
GSH detection via LC-MS in ATCC26933, Δ*egtA*^26933^ and *egtA*^C26933^. (**a**) Extracted Ion Chromatographs (m/z: 695) following LC-MS analysis of TCA precipitated 5′-IAF-labelled mycelial extracts from ATCC26933, Δ*egtA*^26933^ and *egtA*^C26933^, in addition to a GSH standard. A peak at 5.9 min was confirmed to be GSH. (**b**) Peak height data from LC-MS analysis comparing GSH levels in ATCC26933, Δ*egtA*^26933^ and *egtA*^C26933^. GSH levels are significantly (P = 0.0016) increased in Δ*egtA*^26933^ compared to the wild-type and complemented samples. (**c**) Signature Ion breakdown corresponding to 5-IAF labelled GSH.

Supernatants from mycelial lysates following 5′-IAF alkylation and TCA precipitation, from 72 h cultures of ATCC26933, Δ*egtA*^26933^ and *egtA*^C26933^, were analysed via LC-MS. Extracted Ion Chromatograms at *m*/*z* 695 revealed a significantly (P = 0.0016) increased abundance of total cellular glutathione (GSH) in Δ*egtA*^26933^ compared to both the wild-type and complemented strains (Fig. 7 and Supplementary Figure S6). This indicates a significant increase in the level of GSH in response to, or as a result of, abrogation of EGT biosynthesis. LFQ proteomics revealed that cystathionine γ-synthase (CGS; AFUA_7G01590) is missing from Δ*egtA*^26933^. This enzyme converts cysteine to cystathionine and its absence in Δ*egtA*^26933^ may be to ensure cysteine flux is available to facilitate increased GSH biosynthesis (Supplementary Table S1). The ratio of free GSH:GSSG pools increases when *egtA* is deleted, and drops again upon complementation (Supplementary Table S7, Supplementary Figure S6). Thus, absence of EGT biosynthesis is accompanied by an increased relative GSH content in mycelia.

### EGT deficiency increased ferricrocin in wild-type and reduced triacetylfusarinine C but increased fusarinine C content in a ∆*yap1* background

**Figure 8:**
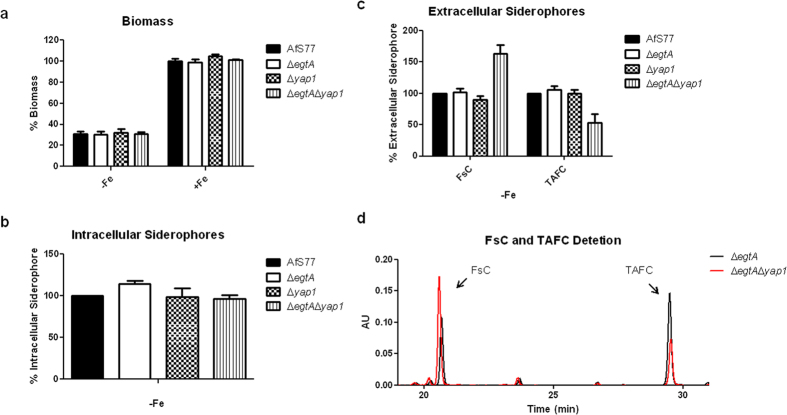
Biomass levels and siderophore production of AfS77, ∆*egtA*^AfS77^, ∆*yap1* and ∆*egtA*∆*yap1* under iron-deplete conditions (containing 20 mM L-glutamine as nitrogen source). (**a**) Biomass production of *A. fumigatus* strains cultivated for 24 h at 37 °C during iron starvation (-Fe) and iron sufficiency (+Fe) show no remarkable differences. (**b**) EGT-deficiency (strain ∆*egtA*) caused higher levels of FC. (**c**) Combined with Yap1 deficiency, EGT deficiency increased FsC and decreased TAFC production (**d**) Exemplar RP-HPLC chromatogram (detection at 435 nm to detect red siderophores after iron saturation of the culture supernatants) confirming increased FsC and decreased TAFC production in ∆*egtA*∆*yap1* (red line) in comparison to ∆*yap1* (black line).

To analyze a possible role of EGT in adaptation to iron starvation, production of biomass and siderophores of strains lacking *egtA, yap1* or both was compared to the wild-type during iron sufficiency and starvation. The biomass production of the strains did not differ significantly (Fig. 8a). Biomass production during iron depletion was about 30% compared to iron sufficiency confirming iron starvation conditions. During iron starvation, *A. fumigatus* produces the intracellular siderophore ferricrocin (FC) and the two extracellular siderophores fusarinine C (FsC) and triacetylfusarinine C (TAFC)^34^. In the wild-type, EGT deficiency increased the cellular accumulation of FC by about 15% (Fig. 8c). In *A. fumigatus* ∆*egtA*∆*yap1*, EGT deficiency increased production of FsC by 60% and decreased production of TAFC by 53%, while the total extracellular siderophore production was not significantly altered. Figure 8d shows an exemplar RP-HPLC analysis of culture supernatants, demonstrating the increase of FsC and decrease of TAFC in the ∆*egtA*∆*yap1* strain compared to ∆*yap1*.

### EGT deficiency leads to transcriptional downregulation of *sidG*

**Figure 9:**
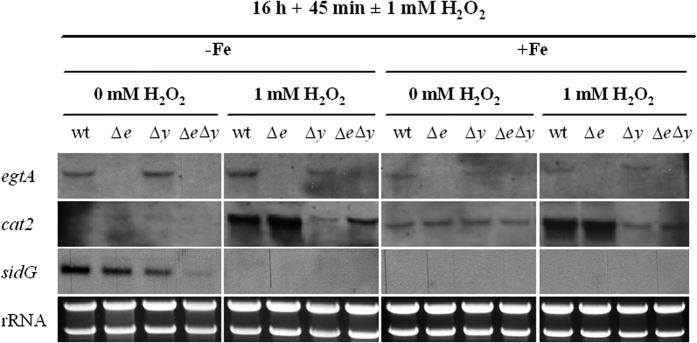
Northern analysis revealing downregulation of *sidG* expression in ∆*egtA*∆*yap1.* Total RNA (10 μg) from AfS77 (wt), ∆*egtA*^AfS77^ (∆*e*), ∆*yap1* (∆*y*) and ∆*egtA*∆*yap1* (∆*e*∆*y*) was isolated from submersed AMM cultures (containing 20 mM L-glutamine as nitrogen source) grown under iron starvation (−Fe) or sufficiency (+Fe) at 37 °C for 16 h plus 45 min with or without addition of H_2_O_2_ to a final concentration of 1 mM. See Supplementary Figure S8.

To further analyze the role of EGT in siderophore production, the transcript levels of selected genes were analysed by Northern analysis during iron starvation and sufficiency with and without H_2_O_2_ stress (Fig. 9). The absence of *egtA* transcripts in ∆*egtA*^AfS77^ confirmed deletion of the gene. Slight up-regulation of *egtA* expression was seen after wild-type treatment with H_2_O_2_, which is in accordance with the results for ATCC26933 exposed to 3 mM H_2_O_2_. The up-regulation of the catalase-peroxidase gene *cat2* (AFUA_8G01670)^35^ in response to H_2_O_2_ treatment in wild-type and ∆*egtA* confirms oxidative stress. The lack of *cat2* up-regulation in *∆yap1* is consistent with the function of Yap1 as an activator of oxidative stress response^28^. The conversion of FsC to TAFC is mediated by the acetyltransferase SidG (AFUA_3G03650)^34^. As shown in Fig. 9, *sidG* is transcriptionally up-regulated during iron starvation, as previously shown^34^, and down-regulated in ∆*egtA*, ∆*yap1* and nearly undetectable in ∆*egtA*∆*yap1*. Notably, *sidG* expression is completely absent after treatment with H_2_O_2_. The down-regulation of *sidG* is consistent with increased FsC and decreased TAFC production.

### Gliotoxin production in Δ*egtA* ^26933^ is significantly reduced compared to wild-type

**Figure 10:**
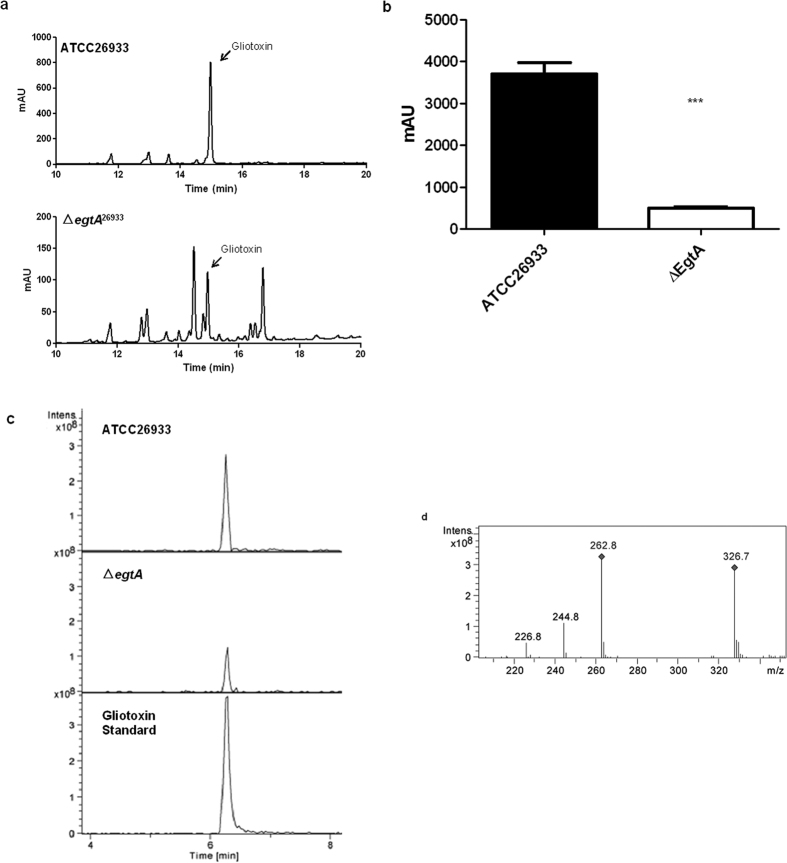
Gliotoxin detection via RP-HPLC and LC-MS in ATCC26933 and Δ*egtA*^26933^. (**a**) RP-HPLC analysis of organic extracts from the supernatants of 72 h cultures of ATCC26933 and Δ*egtA*^26933^. Gliotoxin is present in all chromatograms for both samples at 14.9 min. (**b**) Comparison of gliotoxin peak area from RP-HPLC analysis for ATCC26933 and Δ*egtA*^26933^ performed in triplicate. The peak area was found to be significantly (P = 0.0003) lowered in Δ*egtA*^26933^. (**c**) Extracted Ion Chromatographs (m/z: 327) following LC-MS analysis of organic extracts of supernatants from ATCC26933 and Δ*egtA*^26933^, in addition to a gliotoxin standard. A peak at 6.3 min was confirmed to be gliotoxin. (**d**) Signature ion breakdown corresponding to gliotoxin.

Organic extracts from 72 h culture supernatants of ATCC26933 and Δ*egtA*^26933^ were analysed via RP-HPLC, gliotoxin was detected at a retention time of 14.9 min in both wild-type and Δ*egtA*^26933^ and subsequent analysis via LC-MS confirmed the presence of gliotoxin (Fig. 10). However, gliotoxin levels in *A. fumigatus* Δ*egtA*^26933^ were significantly reduced compared to the wild-type (P = 0.0003) (Fig. 10b). Thus, an inability to biosynthesize EGT appears to lead to attenuated gliotoxin production compared to wild-type. Relevantly, gliotoxin oxidoreductase GliT (AFUA_6G09740), essential for gliotoxin production^36^, was absent from Δ*egtA*^26933^ through LFQ proteomic analysis (Supplementary Table S1), which could, at least in part, explain the significant diminution of gliotoxin biosynthesis in *A. fumigatus* Δ*egtA*^26933^. GliT absence in Δ*egtA*^26933^ could point towards potential sensitivity as per Schrettl *et al.*^36^, however this was not found to be the case (data not shown). LFQ proteomic comparison of Δ*egtA*^26933^ with and without gliotoxin revealed GliT is still induced by gliotoxin exposure, thus no sensitivity is observed (Supplementary Figure S7).

### Δ*egtA* ^26933^ colonies produce paler conidia and show lower levels of conidiation

**Figure 11:**
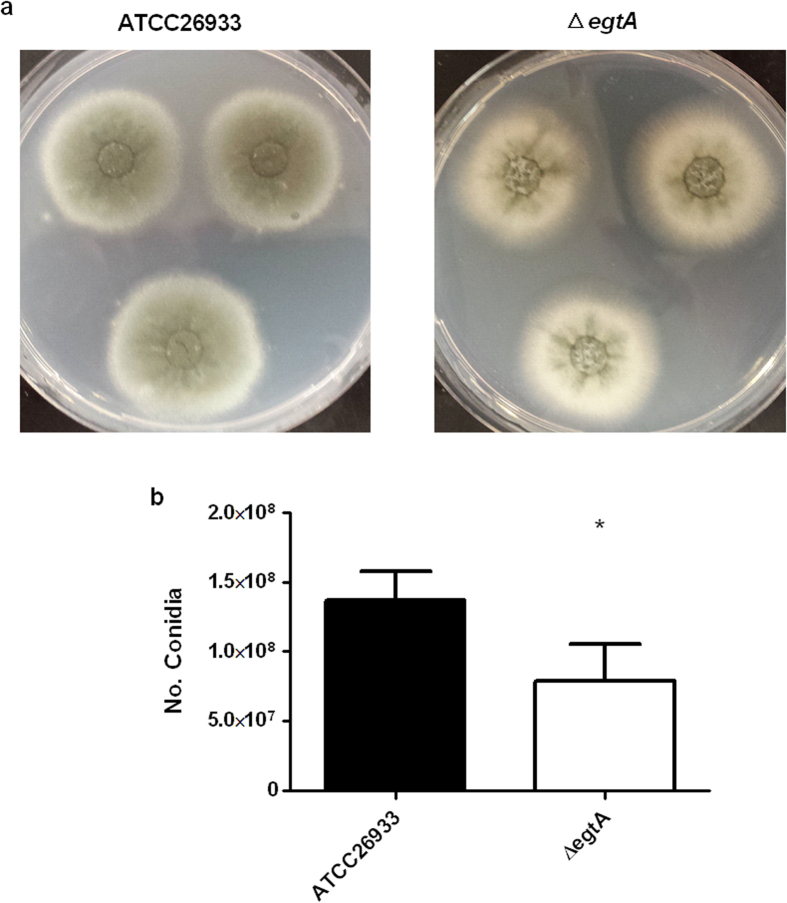
Conidiation in ATCC26933 and Δ*egtA*^26933^. (**a**) Comparison of conidial colour and appearance in colonies from ATCC26933 and *egtA*^26933^. (**b**) Comparison of conidiation levels from ATCC26933 and *egtA*^26933^ (5 mM ammonium tartrate as nitrogen source).

Colonies from Δ*egtA*^26933^ grown on AMM for 72 h exhibited visibly paler conidia compared to those of ATCC26933. Subsequently, levels of conidia were measured by haemocytometry which revealed that Δ*egtA*^26933^ produced significantly (P < 0.05) lower levels of conidia compared to the wild-type (Fig. 11). This suggests a link between EGT and conidial health, as has been previously observed^16^. Relevantly, LFQ proteomics revealed that proteins important for conidiation and conidial health are absent in Δ*egtA*^26933^ (Supplementary Table S1). These include mannosyltransferases PMT2 (AFUA_1G07690) and PMT4 (AFUA_8G04500), conidial hydrophobin RodB (AFUA_1G17250) and FluG (AFUA_3G07140), an extracellular developmental signal biosynthesis protein^35^,^37^,^38^,^39^. In addition, abundance of the LaeA-like protein VipC (AFUA_8G01930) was shown to be increased under basal conditions (log_2_ 1.23 fold increase) and further enhanced when exposed to 3 mM H_2_O_2_ (log_2_ 2.5 fold increase) (Supplementary Tables S1 and S2). VipC is part of the Velvet complex and dysregulation could have consequences for conidiation and has been shown to control the switch between sexual and asexual reproduction in *Aspergillus nidulans*^40^. An *A. nidulans* mutant with over-abundant VipC protein showed reduced asexual development^40^.

## Discussion

Here we present the first identification of the key gene, *egtA*, which encodes EGT biosynthesis in the opportunistic pathogen, *A. fumigatus*. EGT significantly contributes to resistance to high level oxidative stress induced by H_2_O_2_, superoxide and metal ions, and its presence also influences siderophore and gliotoxin biosynthesis. Conidial proteome remodeling occurs in the absence of EGT, which is associated with attenuated conidiation. Overall, we introduce a new player into the sulphur and iron interactome of *A. fumigatus*.

Through Blast searches (http://blast.ncbi.nlm.nih.gov/Blast.cgi), it was shown that the first two enzymes of *M. smegmatis*, EgtD and EgtB, show sequence or functional homologies to a single enzyme in *A. fumigatus*, encoded by AFUA_2G15650, herein termed *egtA*. EgtA from *A. fumigatus* encodes putative sulphatase and methyltransferase activities; additionally it contains an OvoA-domain (5-histidylcysteine sulphoxide synthase domain). In marine organisms and some human pathogens, this enzyme fulfills the function of a sulphoxide synthase catalyzing the first step in ovothiol A biosynthesis to generate an unmethylated sulphur-containing thiohistidine precursor of this metabolite^41^,^42^. The same enzymatic reaction is carried out by EgtB in *M. smegmatis* and by EgtA in *A. fumigatus*. Ovothiol A originates from L-cysteine, L-histidine, O_2_ and SAM, as does EGT^43^,^44^. The iron-dependent step catalyzed by OvoA was described to mediate the oxidative sulphur transfer in *Erwiniata smaniensis*^45^. Ovothiol A is thought to play a role in cellular redox homeostasis in the fertilization of sea urchin eggs^46^, during infection by *Leishmania sp.*, *Trypanosoma sp.*^47^,^48^ and it also induced autophagy in the human hepatic cancer cell line Hep-G2^42^. *In vitro* studies revealed a potent antioxidative function of ovothiols against various radicals *via* ovothiol-promoted NAD(P)H-O_2_ oxidoreductase activity^49^.

The biosynthetic pathway for EGT was recently studied in non-pathogenic fungi. In *S. pombe* the first step is catalyzed by Egt1 leading to hercynylcysteine sulphoxide formation^16^,^17^. The next and last step for the generation of EGT remains still elusive and was not well characterized in eukaryotes. However *S. pombe* likely contains a two-step biosynthetic pathway to generate EGT from L-histidine and utilizes L-cysteine rather than γ-glutamylcysteine as in *M. smegmatis*, since no homolog of *egtC* was found in *S. pombe* which could encode an enzyme capable of glutamyl residue removal^17^. No hits have been found in *A. fumigatus* when searching for homologs of *M. smegmatis* EgtC. However, Pluskal *et al.*^17^ showed that there is a possible homolog of EgtE, a pyridoxal-phosphate (PLP)-binding enzyme in *S. pombe* by screening for EGT production in deletion mutants of possible homologs obtained from a stock center (Bioneer haploid deletion library). They found one mutant strain defective in the gene designated as SPBC660.12c, renamed to *egt2*, which showed lower EGT but raised amounts of the precursor hercynylcysteine sulphoxide. A minimal amount of EGT was still found in the Egt2 deletion strain, explained by a spontaneous conversion of hercynylcysteine sulphoxide to EGT by an unrelated PLP-binding enzyme^17^. While no homologs have been reported for *N. crassa*, a potential homolog to the *M. smegmatis* EgtE and Egt2 from *S. pombe* could be the gene annotated as *lolT* (AFUA_2G13295) in *A. fumigatus*. Further studies will validate this result of sequence homology analysis.

EGT production was abolished in *A. fumigatus* Δ*egtA*^AfS77^ and Δ*egtA*^26933^, and complementation with *egtA* restored EGT production in Δ*egtA*^26933^, thereby confirming the role of the gene in EGT biosynthesis. Both *A. fumigatus* Δ*egtA* deletion strains exhibited a redox-sensitive phenotype at 3 mM H_2_O_2_, with no affects seen at lower H_2_O_2_ concentrations, while wild-type growth was completely inhibited at 4 mM H_2_O_2_. This suggests EGT may act as an auxiliary, or ‘anti-oxidant of last resort’ against oxidative stress in *A. fumigatus*. Sensitivity to menadione was also observed, at both 40 μM and 60 μM, though the mutant matched wild-type growth levels at 20 μM. This reveals that while not essential for primary protection, EGT is important for protection against elevated levels of superoxide radicals in *A. fumigatus*.

Previously, no phenotypic or protective effect of EGT could be shown in *S. pombe* when tested against oxidative stress-inducing agents like H_2_O_2_ and *tert*-butylhydroperoxide^17^ as was demonstrated in *N. crassa*, where EGT contributes to the antioxidative defense against peroxides in conidia and plays a role in conidiogenesis and conidial longevity, but does not protect against UV-induced mutation rate^16^,^50^. However, herein we show that endogenous EGT confers significant resistance against H_2_O_2_ and menadione in the *A. fumigatus*. It was also shown that the key regulator of oxidative response Yap1^28^ is very important to protect *A. fumigatus* against oxidative burden. Additionally, it was demonstrated that EGT deficiency exacerbates the phenotype of the extremely sensitive mutant strain lacking Yap1 too (∆*egtA*∆*yap1*) during treatment with oxidative stressors or metals. The fact that Yap1 deficiency extends the ∆*egtA* phenotype indicates that in wild-type, EGT limitation is partially compensated by other detoxification mechanisms that require activation by Yap1.

The role of EGT in protection against oxidative stress and maintenance of redox homeostasis is further underlined by the results of LFQ proteomics. The abundance of a number of proteins related to redox homeostasis and oxidative stress is dysregulated in Δ*egtA*^26933^ (Supplementary Tables S3 and S4). This apparent proteomic remodelling when Δ*egtA*^26933^ is exposed to H_2_O_2_ underpins the important role played by EGT in maintaining redox homeostasis in the presence of specific oxidants. Loss of EGT results in altered abundance of redox-related proteins, possibly due to an increased level of cellular H_2_O_2_ consequent to abrogation of cellular EGT presence. LFQ proteomic data also suggests that Δ*egtA*^26933^ attempts to synthesise EGT upon H_2_O_2_ exposure, and hints at a possible degradative pathway for EGT. In addition, increased abundance of HisF (AFUA_2G06230) and cystathionine β-lyase (AFUA_4G03950) shows an increased requirement for histidine and SAM respectively, necessary for the biosynthesis of EGT. Furthermore, the increased abundance of a sulphite reductase (AFUA_2G15590) observed could indicate an increased need for sulphur in order to biosynthesise EGT. Previously it has been shown in *A. nidulans* that, the CCAAT-binding complex (CBC, also termed Hap complex or AnCF in *A. nidulans)* senses the cellular redox status via oxidative modification of thiol groups within its subunit HapC^31^. The absence of HapC in Δ*egtA*^26933^ (Supplementary Tables S3 and S4) indicates another layer of CBC regulation, which could lead to an impaired global oxidative stress response.

The changes observed in CGS and CBL in Δ*egtA*^26933^ under basal and ROS conditions when compared to wild-type suggest a metabolic change caused by EGT absence centred on cystathionine usage. Under basal conditions, CGS is downregulated, which would curtail cystathionine production^32^. This would result in cysteine availability for GSH production. Upon addition of 3 mM H_2_O_2_, a “switch” is observed and CBL abundance was increased in Δ*egtA*^26933^ compared to wild-type. This should increase conversion of cystathionine to homocysteine^32^ which may ultimately provide SAM, via the methyl/methionine cycle^51^ for EGT biosynthesis (Fig. 5) and suggests that Δ*egtA*^26933^ attempts to biosynthesise EGT under ROS conditions, thereby highlighting its role in oxidative stress response.

In correspondence, qRT-PCR analysis showed that *egtA* expression was up-regulated in response to H_2_O_2_ exposure, a further indicator that EGT is essential for attenuating oxidative stress in *A. fumigatus*. However despite the increase in *egtA* expression, intracellular EGT levels did not increase upon addition of H_2_O_2_, most likely due to reactivity with ROS species as we observed for purified EGT. This suggests that intracellular EGT is dissipated consequent to its antioxidant activity and is consistent with data from Servillo *et al.*^52^ who reported that EGT is degraded upon oxidation, mainly into hercynine and sulphurous acid. Conversely, the observed rise in EGT levels in Δ*gliK*^18^, deficient in gliotoxin biosynthesis, could be due to either a sensory deficiency in the cellular oxidative stress response, or a compensatory mechanism to replace the frontline antioxidant, GSH, utilised for gliotoxin biosynthesis but which cannot undergo replenishment due to GliK γ-glutamyl cyclotransferase deficiency.

RP-HPLC and LC-MS analysis revealed attenuated gliotoxin production, while GSH production is significantly increased, in Δ*egtA*^26933^. GSH production is likely increased to deal with the increased ROS caused by EGT absence. LFQ proteomic analysis provides revealing insight into the putative mechanisms facilitating this observation. Under basal conditions, cystathionine γ-synthase is undetectable in Δ*egtA*^26933^, which would prevent the conversion of cysteine to cystathionine, therefore channelling more cysteine towards GSH biosynthesis (Fig. 5). GSH is the source of both thiols in gliotoxin^53^,^54^,^55^, and gliotoxin biosynthesis has been shown to be greater in ATCC26933 than ATCC46645^36^. Thus, increased GSH levels in *∆egtA*^26933^ may be consequent to the observed decrease in gliotoxin biosynthesis, and the consequential absence of GSH incorporation into gliotoxin. GSH may, in turn, be diverted from gliotoxin biosynthesis to detoxify ROS, leading to reduced gliotoxin production in Δ*egtA*^26933^. LFQ proteomics also revealed an absence of the gliotoxin oxidoreductase GliT in Δ*egtA*^26933^ (Supplementary Table S6). Deletion of *gliT*, which catalyzes the final step of gliotoxin biosynthesis, results in abrogated gliotoxin production^36^ and its absence in Δ*egtA*^26933^ is entirely in accordance with the observed diminution in gliotoxin biosynthesis. Attenuated gliotoxin production was unexpected, as previous observations in Δ*gliK* suggested an inverse relationship between gliotoxin and EGT. Decreased gliotoxin production following the loss of EGT contradicts that view; however it nonetheless suggests that the production of these two sulphur-containing, redox-active metabolites is interlinked.

Unexpectedly, GSH biosynthesis is not altered in *A. fumigatus* Δ*egtA*^AfS77^ (Supplementary Figure S6). Because of the interconnection of the EGT pathway to GSH biosynthesis in EGT producing bacteria such as *Synechocystis sp.* PCC6803 it had been argued that this interaction may be the case for other EGT-producing organisms, like *A. fumigatus*, resulting in an up-regulation of GSH biosynthesis as a result of a block in the EGT pathway and a higher content of intermediates, or to compensate for the loss of EGT. However, it has been suggested that GSH biosynthesis in EGT-producing fungi does not provide any intermediate for the EGT pathway, because of the missing enzyme annotated as EgtC in *M. smegmatis*, which would catalyze further reactions, remove the glutamyl residue or the absent up-regulation of GSH to utilize the accumulated intermediates or to compensate for the loss of EGT^17^,^56^. Another factor is the previously discussed link between gliotoxin and GSH, the latter essential for the biosynthesis of gliotoxin^53^. With lower gliotoxin production in AfS77 compared to ATCC26933, this may impact the behavior of GSH in the two EGT deficient mutants. Indeed a comparison of GSH levels (Supplementary Figure S6) in the two wild-type strains show that ATCC26933 has lower GSH levels than AfS77, perhaps linked to the different levels of gliotoxin production. The subsequent drop in gliotoxin production in Δ*egtA*^26933^ thus may explain the rise in GSH.

EGT deficiency alters siderophore biosynthesis in *A. fumigatus ∆egtA*^AfS77^. Whereas, FsC and TAFC are produced under iron starvation to be excreted for chelation and import of ferric iron, FC and HFC act as intracellular storage and transport vehicles^22^,^57^. Moreover, it was demonstrated that oxidative stress (H_2_O_2_, PQ) leads to higher levels of FC during both iron states, but most obvious at excess iron in *Aspergillus nidulans* through up-regulation of *sidC*^57^. Siderophore analysis herein implies that EGT is involved in oxidative stress defense because of the higher amounts of FC in EGT-lacking strains. Additionally, this study supports the theory that siderophore biosynthesis is intertwined with oxidative stress response by shifting the production of extracellular siderophores towards the progenitor FsC instead of TAFC in the sensitive ∆*egtA*∆*yap1* mutant strains. Northern analysis related the decreased amounts of TAFC in ∆*egtA*∆*yap1* consequent to a down-regulated expression of *sidG* (AFUA_3G03650), the acetyltransferase catalyzing the triacetylation of FsC to generate TAFC^34^. It was shown previously that HapX deficiency results in selective suppression of TAFC but not FsC biosynthesis, caused by lower transcript levels of *sidG*, but only minor impact on *sidA* and *sidF* expression^58^. This phenotype partially matches that of the ∆*egtA*∆*yap1* mutant indicating that EGT or oxidative stress might negatively influence HapX activity, which leads to the reduction in *sidG* transcription. Alternatively, oxidative stress and EGT, respectively, affect *sidG* sidG expression and HapX independently. Northern blot analysis indicated transcriptional upregulation of *egtA* during iron starvation compared to iron sufficiency (Fig. 9). In this context it is interesting to note that cellular accumulation of the EGT precursor histidine was previously also found to be significantly increased during iron starvation compared to iron sufficiency^58^.

Bello *et al.*^16^ demonstrated that EGT plays an important role in conidial health in *N. crassa*. This seems to be shared with *A. fumigatus*, with paler conidia and lower levels of conidiation observed in Δ*egtA*^26933^. LFQ proteomics reveals several important proteins involved in conidial health and development are missing in Δ*egtA*^26933^ including, conidial hydrophobin RodB (AFUA_1G17250), extracellular developmental signal biosynthesis protein FluG (AFUA_3G07140) and mannosyltransferases PMT2 (AFUA_1G07690) and PMT4 (AFUA_8G04500) (Supplementary Table S1). RodB is a structural protein found in the cell wall of conidia^35^. FluG is a signaling protein associated with asexual development, indeed deletion of FluG in *A. flavus* resulted in reduced conidiation^39^. A mannosyltransferase PMT4 mutant also displayed reduced conidiation, however a deletion mutant of PMT2 was not viable suggesting the gene is essential^38^. Fang *et al.*^37^ demonstrated that reducing *pmt2* transcription in *A. fumigatus* resulted in reduced conidiation, retarded germination and impaired cell wall integrity. The absence of these conidiation-associated proteins suggests that EGT presence, or global cellular redox homeostasis play an important role in the conidiation process in *A. fumigatus*. Moreover, it has been postulated that ROS may play a role in regulating cellular differentiation^59^ and the loss of EGT, and subsequent dysregulation of the redox system, may further explain the loss of these proteins and the effects on conidiation observed in Δ*egtA*^26933^. Another protein with altered expression was VipC (AFUA_8G01930), a methyltransferase that forms part of the Velvet complex^40^ (Supplementary Tables S1 and S2). Sarikaya-Bayram *et al.* demonstrated in *A. nidulans* that VipC is important for the appropriate activation of either sexual or asexual reproduction in response to darkness or light respectively. While increased abundance of VipC was associated with increased asexual reproduction, a VipC over-expression mutant showed repression of both asexual conidiation and sexual fruiting bodies.

To conclude, EGT biosynthesis is linked to multiple, apparently unrelated systems in *A. fumigatus*. Cystathionine metabolism and the transsulphuration pathway is altered in Δ*egtA*, especially under oxidative stress conditions. This ‘cystathionine switch’ could be an important control mechanism to deal with altered redox homeostasis in *A. fumigatus*.

## Methods

### Strains, growth conditions, and oligonucleotides

*A. fumigatus* strains were grown at 37 °C using *Aspergillus* minimal medium (AMM) agar. AMM contained 1% (w/v) glucose as the carbon source, 5 mM ammonium tartrate or 20 mM L-glutamine as the nitrogen source and trace elements^60^. Liquid cultures were performed with 100 ml Czapek-Dox broth or AMM in 500 ml Erlenmeyer flasks inoculated with 10^8^ conidia. For growth assays, 5 × 10^4^ conidia of the respective strains were point inoculated on AMM agar plates containing the relevant supplements and incubated for 72 h at 37 °C. All *A. fumigatus* strains and oligonucleotides used are listed in Supplementary Tables S8 and S9 respectively.

### Deletion of *egtA* and *yap1* and complementation of the ∆*egtA* and ∆*egtA*∆*yap1* strains

For generating *egtA* and *yap1* deletions in AfS77, and the associated double mutants, the bipartite marker technique was used^61^. Briefly, *A. fumigatus* was co-transformed with two DNA fragments, each containing overlapping but incomplete fragments of a resistance cassette (pyrithiamine, *ptrA*; hygromycin, *hph*) fused to the 5′ and 3′ flanks of *egtA*. The *egtA* 5′-flanking region (1199 bp) was PCR amplified from genomic DNA using primers oAfDUF323.1 and oAfDUF323.2. For the amplification of the 3′-flanking region (1149 bp) primers oAfDUF323.3 and oAfDUF323.4 were employed. Subsequent to gel purification, these fragments were digested with *Hind*III and *Pst*I, respectively. The *ptrA* selection marker was released from plasmid pSK275 by digestion with *Hind*III (5′-flanking region) and *Pst*I (3′-flanking region) and ligated with the 5′- and 3′-flanking regions respectively. The transformation construct A (2022 bp, fusion of the *egtA* 5′-flanking region to *ptrA* split marker) was amplified from the ligation product using oAfDUF323.5 and oAoptrA1. For amplification of transformation construct B (2476 bp, fusion of *ptrA* split marker with 3′-flanking region) primers oAoptrA.2 and oAfDUF323.6 were employed. For transformation of *A. fumigatus* protoplasts both constructs were simultaneously used. To generate a knockout mutant strain deficient in Yap1 in the background of AfS77, genomic DNA including the resistance cassette gene *hph* of ∆*yap1* in the genetic background of the AfS77 was amplified using primers oAfyap1.5′f and oAfyap1.3′r (4 kb). The PCR-product was transformed in *A. fumigatus* AfS77 and ∆*egtA* protoplasts to generate ∆*yap1* and ∆*egtA*∆*yap1*, respectively.

The same strategy^61^ was used for the generation of Δ*egtA*^26933^. *A. fumigatus* strain ATCC26933 was co-transformed with two DNA constructs, both containing incomplete fragments of the pyrithiamine gene, *ptrA*^62^. Fragments were generated via PCR from DNA extracted from Δ*egtA*^AfS77^ using primers oAFDUF323.5 and oAoPtrA1 for the 5′ fragments, and oADUF323.6 and oAoPtrA2 for the 3′ fragment. Generation of the mutant was confirmed by Southern analysis^53^.

For the reconstitution of Δ*egtA*^AfS77^, a functional copy of *egtA* (a 5691 bp PCR-amplified fragment generated with the primers oAetgA1.7_f and oAegtA1.8_r) was subcloned into pAN8.1 containing the phleomycin resistance cassette (*ble*) by a digestion with *Sph*I and *Nhe*I resulting in *(p)egtA:ble*. The resulting 10,508 bp plasmid was linearized with *Sph*I and used for the transformation of *A. fumigatus* protoplasts of ∆*egtA* and ∆*egtA*∆*yap1*. To complement Δ*egtA*^26933^, a PCR fragment containing the *egtA* locus including promoter and terminator was amplified using primers oAfDUF323.5 and oAfDUF323.6 and then inserted into the pCR^®^ 2.1-TOPO^®^ TA vector. This vector (*pegtA*) was linearised with *Aat*II and transformed into Δ*egtA*^26933^ alongside the plasmid pAN 7-1, containing the *hph* selection marker for hygromycin resistance. Insertion of the *egtA* gene was confirmed via Southern analysis. Transformation of *A. fumigatus* was carried out as described previously^25^. For selection of positive transformants 0.1 mg/ml pyrithiamine (Sigma), 0.2 mg/ml hygromycin (Sigma) or 0.02 mg/ml phleomycin (Eubio) was used. Southern blot analyses were used to screen for positive transformants. PCR primers used for generating hybridization probes are listed in Supplementary Table S9.

### RNA Isolation, Reverse Transcription PCR and Quantitative PCR

RNA was isolated and purified from *A. fumigatus* hyphae crushed in liquid N_2_ using the RNeasy plant minikit (Qiagen). RNA was treated with DNase 1 (Invitrogen), and cDNA synthesis from mRNA (500 ng) was performed using a qScript cDNA SuperMix kit (Quanta Biosciences). The gene encoding calmodulin (AFUA_4G10050)^63^, which is constitutively expressed in *A. fumigatus*, served as a control in RT-PCR and RT-qPCR experiments. RT-qPCR was performed using a Roche Lightcycler 480^64^. Northern Analysis was performed as described elsewhere^24^.

### Ergothioneine Analysis

To analyze ergothioneine production, *A. fumigatus* ATCC26933, Δ*egtA*^26933^ and *egtA*^C26933^ were grown at 37 °C for 72 h in Czapek-Dox broth. Mycelia were snap frozen in liquid N_2_ and lyophilised overnight. Lyophilised mycelia were bead beaten in lysis buffer, incubated on ice for 1 h and centrifuged (13,000 g) at 4 °C. Supernatants (50 μl) were treated with 5′-IAF (10 μl; 3 mg/ml in DMSO) and analyzed via RP-HPLC and LC-MS as described^18^.

### Sensitivity Assays for Oxidative Stress

*A. fumigatus* strains were incubated on AMM agar at 37 °C for up to 72 h in the presence of oxidising agents. Sensitivity was determined by comparing mean radial growth from 3 replicates, significance was determined via one-way ANOVA. Oxidising agents used were H_2_O_2_ (0–3 mM), menadione (0–60 μM) and diamide (0–1.75 mM). Metal toxicity was assayed with iron (0–10 mM), copper (0–1.0 mM), zinc (0–8 mM), and cobalt (0–1 mM).

### Determination of Ergothioneine reactivity with H_2_O_2_

Reactivity between EGT and H_2_O_2_ was analysed by incubating 3 mM EGT (Sigma-Aldrich) with, and without, 3 mM H_2_O_2_ for 3 h at room temperature. Triplicate specimens were then analyzed by back-titration to detect remaining EGT by labelling with 5′-IAF followed by RP-HPLC detection as described above. Residual EGT amounts were compared using unpaired t-test.

### Glutathione Analysis via LC-MS

*A. fumigatus* mycelia were grown for 72 h at 37 °C in Czapek-Dox broth, snap-frozen in liquid N_2_ and lyophilised overnight. Lyophilised mycelia were bead beaten in lysis buffer, incubated on ice for 1 h and centrifuged (13,000 g) at 4 °C. Supernatants (50 μl) were treated with 5′-IAF (10 μl; 3 mg/ml in DMSO) and analyzed using LC-MS as described^18^. GSH levels were compared using one-way ANOVA.

#### GSH/GSSG determination

*A. fumigatus* mycelia were grown for 72 h at 37 °C in Czapek-Dox Broth, harvested through miracloth and snap frozen in liquid N_2_. GSH and GSSG levels were then determined as described previously^65^.

### Comparative Label Free Quantitative (LFQ) Proteomic Analysis of *A. fumigatus* ATCC26933 and Δ*egtA* ^*26933*^

*A. fumigatus* ATCC26933 and Δ*egtA*^26933^ (*n* = 4 biological replicates each) were cultured in Sabouraud-Dextrose media for 23 h followed by H_2_O_2_ addition (3 mM final) or equivalent volume of H_2_O for 1 h. Mycelial lysates were prepared in lysis buffer (100 mM Tris-HCl, 50 mM NaCl, 20 mM EDTA, 10% (v/v) Glycerol, 1 mM PMSF, 1 μg/ml pepstatin A, pH 7.5) with grinding, sonication and clarified using centrifugation (13,000 g, 20 min, 4 °C). Lysates were then precipitated using trichloroacetic acid/acetone and resuspended in 100 mM Tris-HCl, 6 M urea, 2 M thiourea, pH 8.0. Samples were reduced and alkylated using DTT and iodoacetamide respectively, then treated with trypsin and ProteaseMax surfactant^55^,^66^. Resultant peptide mixtures were analyzed via a Thermo Fisher Q-Exactive mass spectrometer coupled to a Dionex RSLCnano. LC gradients operated from 4 to 45% B over 2 h, and data was collected using a Top15 method for MS/MS scans. Comparative proteome abundance and data analysis were performed using MaxQuant software (version 1.3.0.5^67^), with Andromeda used for database searching and Perseus used to organize the data (version 1.4.1.3).

### Siderophore analysis

Analysis of extracellular and intracellular siderophores was performed by RP-HPLC as described previously^24^.

### Gliotoxin Analysis

To analyze gliotoxin production, *A. fumigatus* ATCC26933 and Δ*egtA*^26933^ were grown at 37 °C for 72 h in Czapek-Dox medium. Supernatants were chloroform extracted and fractions were dried to completion under vacuum. Extracts were resolubilised in methanol and analyzed using RP-HPLC and LC-MS as previously described^36^. Gliotoxin levels were compared via unpaired t-test.

### Conidial Quantification

Colonies of ATCC26933 or Δ*egtA*^26933^ were grown on AMM (containing 5 mM ammonium tartrate as nitrogen source) for 72 h at 37 °C. Plugs were taken from the centre of each colony and placed into 1 ml of PBS in 1.5 ml tubes. After vigorous vortexing, 20 μl of each solution was placed on a haemocytometer and quantified. Mean conidial concentrations were computed from *n* = 3 independent colonies. Conidial concentrations were compared via unpaired t-test.

## Additional Information

**How to cite this article**: Sheridan, K. J. *et al.* Ergothioneine Biosynthesis and Functionality in the Opportunistic Fungal Pathogen, *Aspergillus fumigatus. Sci. Rep.* **6**, 35306; doi: 10.1038/srep35306 (2016).

## Supplementary Material

Supplementary Dataset 1

Supplementary Dataset 2
