# Supplementary material for: Ergothioneine Biosynthesis and Functionality in the Opportunistic Fungal Pathogen, Aspergillus fumigatus
Source: Sci Rep. 2016 Oct 17;6:35306. doi: 10.1038/srep35306 (PMC5066259; doi:10.1038/srep35306)
Supplement: Supplementary Dataset 1 [file srep35306-s1.doc]

**­­Running head:***A. fumigatus* ergothioneine.

**Ergothioneine Biosynthesis and Functionality in the Opportunistic Fungal Pathogen, *Aspergillus fumigatus*.**

Kevin J. Sheridan^1^*, Beatrix Elisabeth Lechner^2^*, Grainne O’Keeffe^1^, Markus A. Keller^3^, Ernst R. Werner^3^, Herbert Lindner^4^, Gary W. Jones^1^, Hubertus Haas^2^,^, Sean Doyle^1^,^.

^1^Department of Biology, Maynooth University, Maynooth, Co. Kildare, Ireland.

^2^Division of Molecular Biology, Biocenter, Medical University Innsbruck, Innrain 80/82, Austria.

^3^Division of Biological Chemistry and ^4^Division of Clinical Biochemistry, Biocenter, Medical University Innsbruck, Innrain 80/82, Austria.

* Equal contribution.

**^ Joint corresponding authors.**

Professor Sean Doyle, Department of Biology, Maynooth University, Maynooth, Co. Kildare, Ireland. Tel: +353-1-7083858; E-mail: [sean.doyle@nuim.ie](mailto:sean.doyle@nuim.ie); Web: [http://biology.nuim.ie](http://biology.nuim.ie/)

Professor Hubertus Haas Division of Molecular Biology, Biocenter, Medical University Innsbruck, Innrain 80/82, Austria. Tel: +43 512 9003 70205; Email: [hubertus.haas@i-med.ac.at](mailto:hubertus.haas@i-med-ac.at); Web: <http://mol-biol.i-med.ac.at/staff/h_haas.html>

**Table S3:** Proteins from LFQ-proteomics associated with oxidative stress and redox homeostasis with significant Log_2_ fold change or unique/absent in *A. fumigatus* ∆*egtA*^26933^compared to ATCC26933 under basal conditions. Data sorted by fold change, in descending order.

| **Protein Description** | **Present/**  **Log_2_**  **(Fold Change)** | **p value** | **Peptides** | **Sequence**  **coverage**  **[%]** | **Protein IDs** |
| --- | --- | --- | --- | --- | --- |
| 2-dehydropantoate 2-reductase | 1.48361 | 0.018129 | 5 | 16.7 | AFUA_4g13960 |
| Sulfhydryl oxidase | 1.22122 | 0.008832 | 7 | 37.3 | AFUA_3g08850 |
| Oxidoreductase, 2OG-Fe(II) oxygenase family | 1.10977 | 0.018011 | 16 | 58.7 | AFUA_1g01000 |
| Oxidoreductase, short chain dehydrogenase/reductase family | -1.0044 | 0.00741 | 13 | 86.4 | AFUA_5g14000 |
| Cytochrome P450 monooxygenase, putative | -1.01068 | 0.030958 | 15 | 34.7 | AFUA_6g02210 |
| 5-oxo-L-prolinase, putative | -1.08559 | 0.000788 | 65 | 63.9 | AFUA_6g14330 |
| Isoflavone reductase family protein (Oxidoreductase activty) | -1.09131 | 0.021158 | 13 | 57 | AFUA_1g12510 |
| Mitochondrial enoyl reductase, putative | -1.23179 | 0.001484 | 17 | 61.8 | AFUA_3g03330 |
| Oxidoreductase, zinc-binding dehydrogenase family, putative | -2.01615 | 0.02697 | 12 | 61 | AFUA_1g15610 |
| Cystathionine gamma-synthase | Absent | n/a | 7 | 30.6 | AFUA_7g01590 |
| Cytochrome c oxidase assembly protein (Pet191), putative | Absent | n/a | 2 | 26.5 | AFUA_5g08965 |
| FAD dependent oxidoreductase, putative | Absent | n/a | 7 | 27.2 | AFUA_6g04220 |
| Tartrate dehydrogenase, putative | Absent | n/a | 6 | 22.3 | AFUA_1g04150 |
| MAP kinase kinase (Mkk2), putative | Absent | n/a | 2 | 5.8 | AFUA_1g05800 |
| Probable 4-hydroxyphenylpyruvate dioxygenase 2 | Absent | n/a | 6 | 24.1 | AFUA_4g10620 |
| NADH-ubiquinone oxidoreductase 64 kDa subunit, putative | Absent | n/a | 6 | 11.4 | AFUA_2g05450 |
| FAD-dependent oxygenase, putative | Absent | n/a | 4 | 10.3 | AFUA_3g00840 |

**Table S4:** Proteins from LFQ-proteomics associated with oxidative stress and redox homeostasis with significant Log_2_ fold change or unique/absent in *A. fumigatus* ∆*egtA*^26933^compared to ATCC26933 under oxidative stress conditions. Data sorted by fold change, in descending order.

| **Protein Description** | **Present/**  **Log_2_**  **(Fold Change)** | **p value** | **Peptides** | **Sequence**  **coverage**  **[%]** | **Protein IDs** |
| --- | --- | --- | --- | --- | --- |
| NADPH-dependent FMN reductase Lot6, putative | 4.40163 | 0.00432 | 8 | 69.2 | AFUA_7g06600 |
| Pyridoxal kinase, putative | 3.61996 | 0.007665 | 7 | 34.6 | AFUA_1g02900 |
| NmrA-like family protein | 3.19014 | 0.010049 | 8 | 44.7 | AFUA_6g00280 |
| Cystathionine beta-lyase MetG | 3.09553 | 0.0355343 | 8 | 32.5 | AFUA_4g03950 |
| Oxidoreductase, 2OG-Fe(II) oxygenase family | 2.88667 | 0.002938 | 18 | 60 | AFUA_1g01000 |
| Short chain dehydrogenase family protein | 2.87187 | 0.011169 | 6 | 23 | AFUA_1g16630 |
| CsgA-like short chain dehydrogenase/reductase, putative | 2.86184 | 0.046537 | 6 | 62.8 | AFUB_075540 |
| NifU-related protein | 2.62307 | 0.048628 | 7 | 44.8 | AFUA_1g04680 |
| Flavin-binding monooxygenase-like protein | 2.56424 | 0.027902 | 8 | 21.2 | AFUA_6g01900 |
| Ortholog(s) have cytochrome-c oxidase activity | 2.50787 | 0.028945 | 5 | 21.3 | AFUA_Mt00120 |
| Short-chain dehydrogenase, putative | 2.45341 | 0.019904 | 7 | 29.3 | AFUA_8g00280 |
| Sulfite reductase, putative | 2.22575 | 0.003046 | 35 | 31.2 | AFUA_2g15590 |
| NADH-ubiquinone oxidoreductase subunit GRIM-19, putative | 2.20179 | 0.038593 | 4 | 34.6 | AFUA_3g08770 |
| Aflatoxin B1-aldehyde reductase GliO-like, putative | 2.01845 | 0.049263 | 13 | 48.3 | AFUA_1g13370 |
| DUF255 domain protein | 1.88685 | 0.041084 | 12 | 20.8 | AFUA_1g12370 |
| 2-dehydropantoate 2-reductase | 1.80192 | 0.041835 | 4 | 16.1 | AFUA_3g00740 |
| 2-oxo acid dehydrogenases acyltransferase, putative | 1.62889 | 0.037992 | 17 | 46.3 | AFUA_4g12010 |
| NADH-ubiquinone oxidoreductase 304 kDa subunit | 1.50531 | 0.043681 | 16 | 52.1 | AFUA_6g08810 |
| AhpC/TSA family protein | 1.44847 | 0.014711 | 11 | 54.1 | AFUA_6g12500 |
| NADH-dependent flavin oxidoreductase, putative | 1.43235 | 0.030737 | 33 | 76.1 | AFUA_7g06420 |
| Pyridoxal reductase (AKR8), putative | 1.42837 | 0.01439 | 11 | 38.6 | AFUA_1g10270 |
| Glycerol dehydrogenase (GldB), putative | 1.37995 | 0.019009 | 30 | 85.5 | AFUA_4g11730 |
| Arsenate reductase (Arc2), putative | 1.27464 | 0.027714 | 9 | 63.3 | AFUA_6g13400 |
| 2-methylcitrate dehydratase, putative | 1.23478 | 0.009205 | 38 | 65.2 | AFUA_6g03730 |
| DNA damage-inducible protein 1 | 1.21829 | 0.034152 | 24 | 73.8 | AFUA_7g06050 |
| Short chain dehydrogenase/reductase family protein | 1.17302 | 0.024341 | 13 | 80.5 | AFUA_1g00990 |
| Imidazole glycerol phosphate synthase subunit hisF | 1.10807 | 0.024405 | 32 | 74.4 | AFUA_2g06230 |
| Pyridoxamine phosphate oxidase, putative | 1.13607 | 0.038128 | 17 | 52.4 | AFUA_5g10650 |
| Flavin containing amine oxidase, putative | Unique | n/a | 3 | 9.3 | AFUA_3g12150 |
| Cytochrome P450, putative | Unique | n/a | 1 | 4 | AFUA_5g01360 |
| Autophagy protein Atg20, putative | Unique | n/a | 3 | 7.5 | AFUA_2g14160 |
| Epoxide hydrolase, putative | Unique | n/a | 4 | 19 | AFUA_2g16900 |
| Protein kinase Yak1, putative | Unique | n/a | 4 | 5.5 | AFUA_4g03850 |
| Zinc-binding oxidoreductase CipB | Unique | n/a | 6 | 28.8 | AFUA_4g00700 |
| Aldehyde dehydrogenase, putative | -1.07759 | 0.046538 | 24 | 53.7 | AFUA_2g00720 |
| Cytochrome B | -1.13698 | 0.017889 | 2 | 4.9 | AFUA_Mt00001 |
| Trimethyllysine dioxygenase TmlH, putative | -1.27237 | 0.027847 | 6 | 25.2 | AFUA_1g06180 |
| Glycerol kinase, putative | -1.99179 | 0.048473 | 7 | 20.7 | AFUA_4g11540 |
| Ferrooxidoreductase Fet3, putative | Absent | n/a | 2 | 5.6 | AFUA_5g03790 |
| Oxidoreductase CipA-like, putative | Absent | n/a | 3 | 13.4 | AFUA_1g12460 |

**Table S7:** GSH/GSSG ratios of the two wild type strains, *egtA* deletion mutants and *egtA* complemented strains.

| **Strain** | **GSH/GSSG Ratio** |
| --- | --- |
| ATCC26933 | 2.7 |
| Δ*egtA*^26933^ | 3.16 |
| *egtA*^C26933^ | 1.52 |
| AfS77 | 1.4 |
| Δ*egtA*^AfS77^ | 3.74 |
| *egtA*^CAfS77^ | 0.64 |

**Table S8:** *A. fumigatus* strains used in this study.

| **Strain** | **Genotype** | **Reference** |
| --- | --- | --- |
| ATCC26933 | Wild-Type | http://www.atcc.org/ |
| AfS77 | ATCC46645, ∆akuA::loxP | Hartmann et al., 2010 |
| Δ*egtA*^26933^ | ATCC26933, Δ*egtA* | This Work |
| Δ*egtA*^AfS77^ | AfS77, Δ*egtA::ptrA* | This Work |
| ∆*egtA*∆*yap1* | AfS77, ∆*egtA::ptrA;* ∆*yap1::hph* | This Work |
| *egtA*^C26933^ | ATCC26933, ∆*egtA:egtA* | This Work |
| *egtA*^CAfS77^ | AfS77, Δ*egtA:egtA; ble* | This Work |
| *egtA*^C^∆*yap1* | AfS77, ∆*egtA*∆*yap1*::*egtA; ble* | This Work |

**Table S9:** List of oligonucleotides used in this study.

| **Primer Name** | **Sequence (5' to 3')** |
| --- | --- |
| oAfDUF323.1 | GATCCACAAGATACGGGC |
| oAfDUF323.2 | GGTGGGCAATGATCTTGG |
| oAfDUF323.3 | CCGTCTTTGGACCTGTTC |
| oAfDUF323.4 | GATAGTCTCCGCCGAAAG |
| oAfDUF323.5 | TCATCCATCTCGCCTCTG |
| oAfDUF323.6 | CAGGGTACATCCAGGTAC |
| oAetgA1.7_f | AGGACTGTTACAGGCGAG |
| oAegtA1.8_r | ATCTGCTCCGTCAACTGG |
| oAfyap1.5’f | TTGTGAACCGCTCCATCG |
| oAfyap1.3’r | TGAGCGAGATGCACATGG |
| oAoPtrA1 | GAGGACCTGGACAAGTAC |
| oAoPtrA2 | CATCGTGACCAGTGGTAC |
| Q DUF323 F | CTATGATGATGCCCTTGCCT |
| Q DUF323 R | GCATTCGCATGTAATAGCCC |
| CGS QPCR F | AGGATTGGGCGAAGAAACTC |
| CGS QPCR R | CACACCAAGCCAACTCTCCA |
| CBL QPCR F | TGAACGACCTTGCTCTTGGG |
| CBL QPCR R | GAGCCAGGAGTCAAACGGAG |


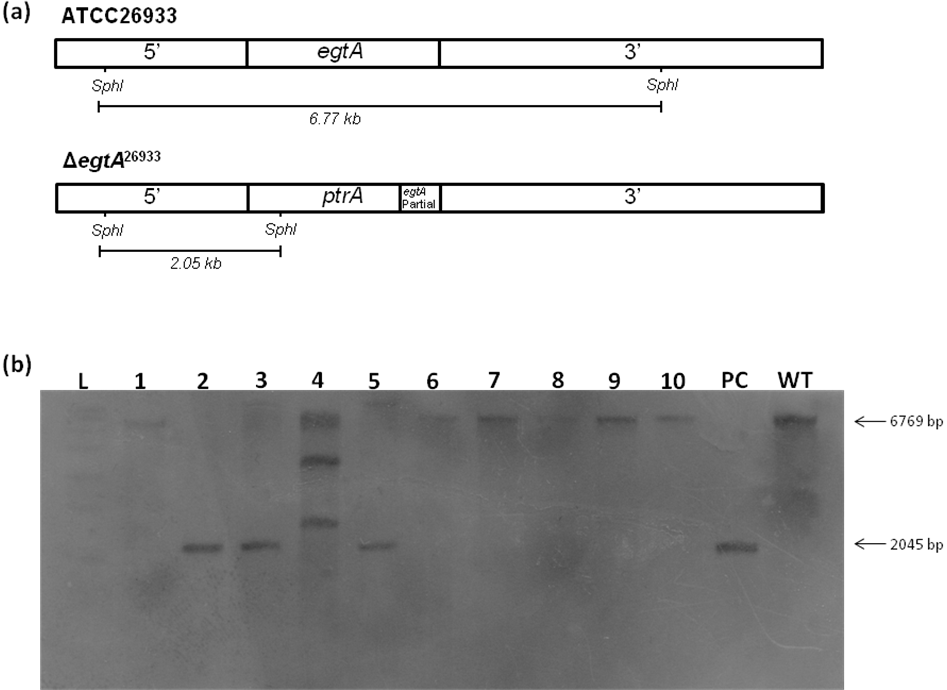


**Figure S1:**Δ*egtA*^26933^ Southern blot (a) Southern strategy for ATCC26933 and Δ*egtA* (b)Southern Analysis of potential *egtA* knock out mutants in *A. fumigatus* strain ATCC26933 using a 5’ probe. Lane L: Molecular weight marker ladder (Roche VII, DIG labelled), Lane 1-9: Possible transformants, Lane PC: Δ*egtA*^46645^, Lane WT: ATCC26933. Wild type genotype displays a band at 6.8 kb (Lane WT). Δ*egtA* genotype displays a band at 2 kb (Lane PC). Transformant 2 displays the correct band for the *egtA* genotype.


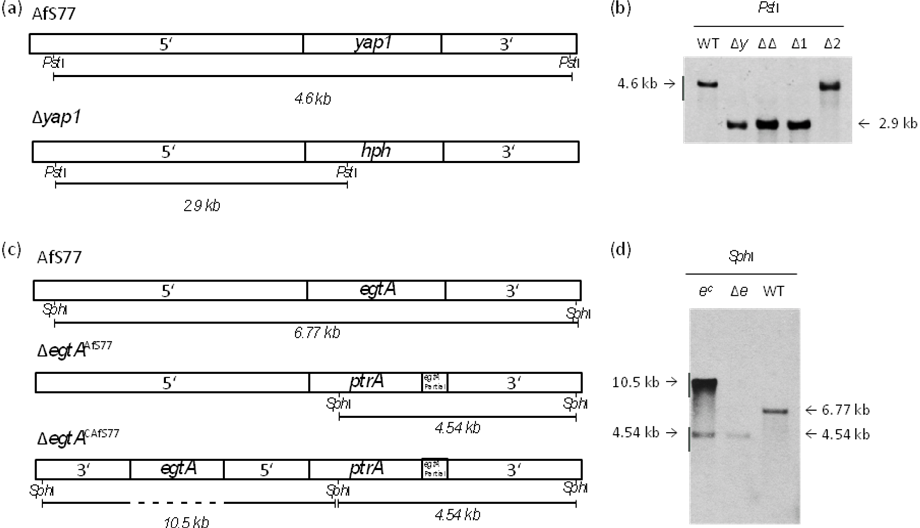


**Figure S2:**(a) Southern strategy for AfS77 (WT), ∆*yap1* (∆), ∆*egtA*∆*yap1* (∆∆). (b) Southern analysis of *yap1* (∆1, ∆2)and ∆*yap1*∆*egtA* (∆∆)strains in *A. fumigatus* strain AfS77 using a 5’ probe. Lane WT: AfS77, Lane ∆*y*: ∆*yap1* (ATCC46645), Lane ∆∆: ∆*yap1*∆*egtA*, Lane ∆1, ∆2: ∆*yap1* (AfS77) transformants. Wild-type genotype displays a band at 4.6 kb (Lane WT). *Δyap1*genotype displays a band at 2.9kb (Lane Δ*y*, ∆∆ and ∆1). Transformant ∆∆ and ∆1 display the correct band for the ∆*yap1*genotype (2.9 kb). (c) Southern strategy for AfS77 (WT),Δ*egtA*^AfS77^ (∆*e*) and *egtA^c^*∆*yap1* (*e^c^*). (d) Southern analysis of an *egtA* complemented strain in *A. fumigatus* strain AfS77 using a 3’ probe. Lane *e^c^*: *egtA*^CAfS77^ transformant, Lane Δ*e*: Δ*egtA*^AfS77^; Lane WT: AfS77. Wild-type genotype displays a band at 6.77 kb (Lane WT). Δ*egtA* genotype displays a band at 4.54kb (Lane Δ*e*). Complemented *egtA* in ∆*egtA*∆*yap1* displays a band at 4.54 kb and 10.5 kb (Lane *e^c^*), containing both the Δ*egtA* and *egtA*^C^ bands.


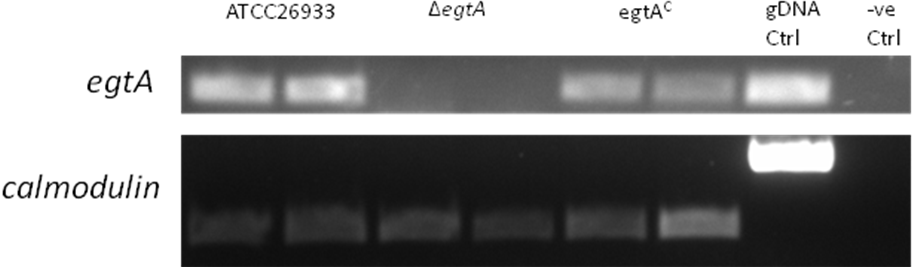


**Figure S3:** 1% agarose gel, showing the results from RT-PCR for *egtA* gene expression in ATCC26933 Δ*egtA*^26933^ and *egtA*^C^. Also included are a genomic DNA control and a negative control.*calm* gene expression for the replicates from all 3 samples, as well as the gDNA control, shows expected expression. For *egtA*, expression is observed in the wild type, *egtA*^C^ and gDNA samples but is absent in the knock out strain.


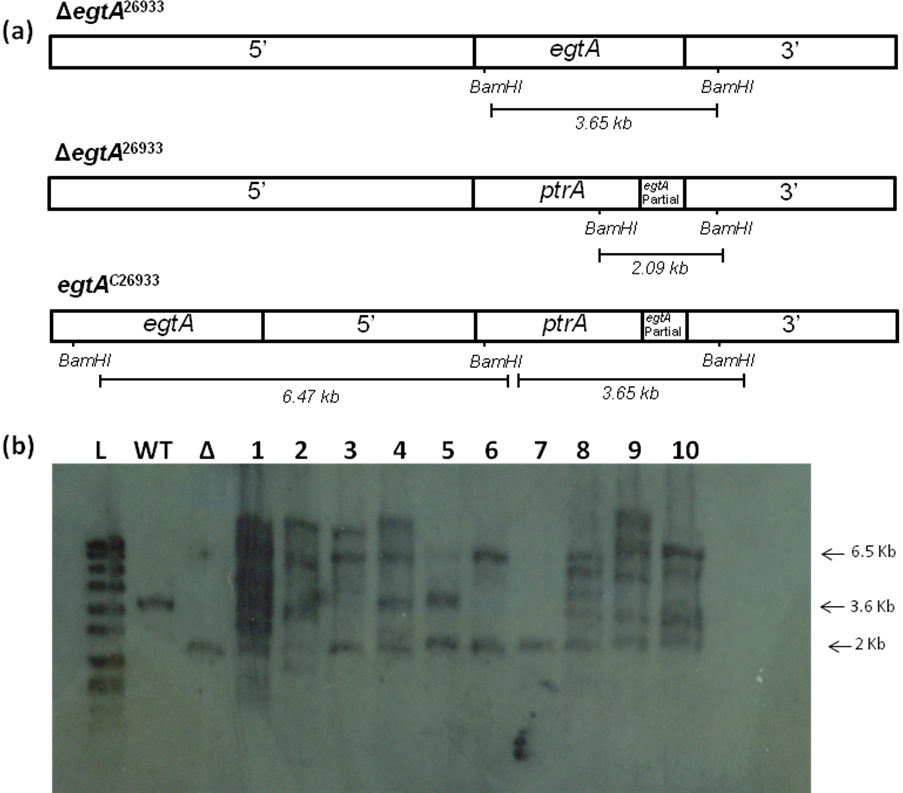


**Figure S4:***egtA*^C26933^ Southern blot. (a) Southern strategy for ATCC26933, Δ*egtA* and *egtA*^C^. (b) Southern Analysis of potential *egtA* complemented strains in *A. fumigatus* strain ATCC26933 using a 3’ probe. Lane L: Molecular weight marker ladder (Roche VII, DIG labelled), Lane WT: ATCC26933, Lane Δ: Δ*egtA*^26933^, Lane 1-10: Potential *egtA*^C^ transformants. Wild-type genotype displays a band at 3.6 kb (Lane WT). Δ*egtA* genotype displays a band at 2 Kb (Lane Δ). Complented *egtA* displays a band at 6.5 Kb. Transformant 6 displays the correct bands for the *egtA*^C^genotype, containing both the Δ*egtA* and *egtA*^C^  bands at 2 kb and 6.5 kb respectively.


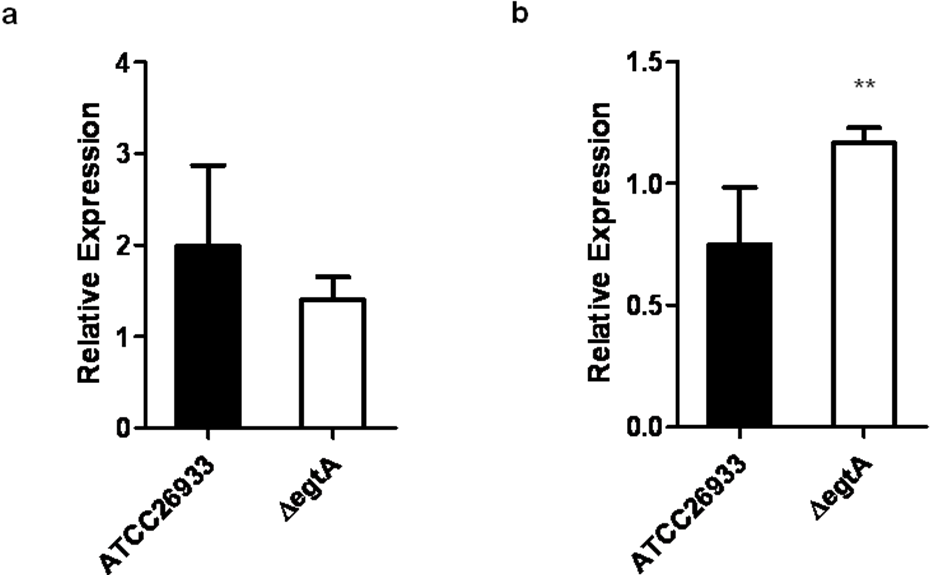


**Figure S5:** RT-qPCR data for CGS and CBL. (a) RT-qPCR data for CGS in ATCC26933 and Δ*egtA*^26933^ under basal conditions. A non-significant drop in CGS expression is observed in Δ*egtA*^26933^ compared to ATCC26933. (b) RT-qPCR data for CBL in ATCC26933 and Δ*egtA*^26933^ following 3 mM H_2_O_2_ exposure for 1 h. A significant (P = 0.003) rise in CBL expression is observed in Δ*egtA*^26933^ compared to ATCC26933.


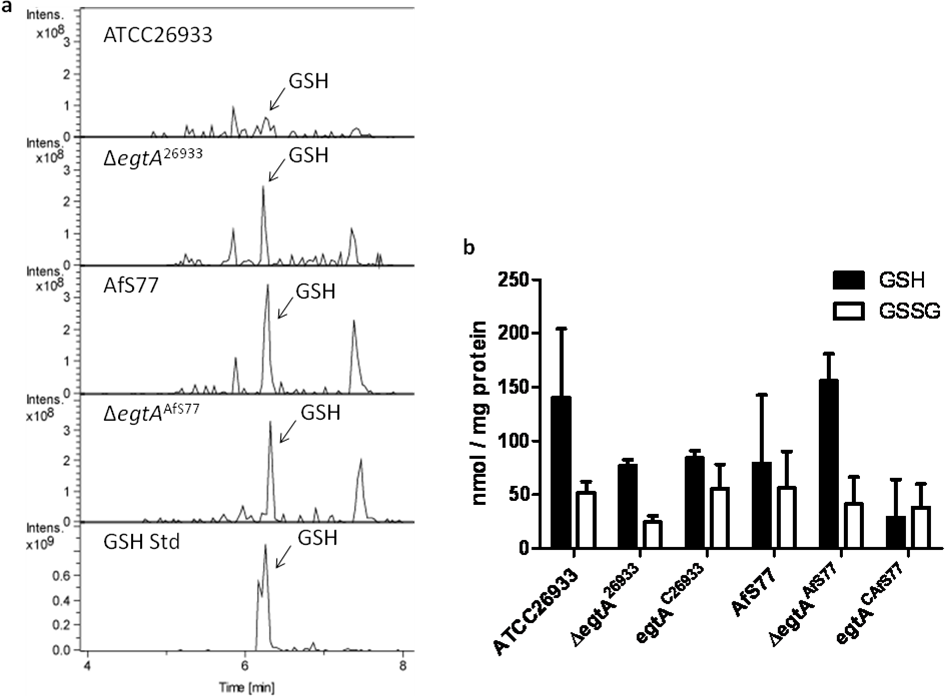
**Figure S6:** GSH in different backgrounds. (a) Extracted Ion Chromatographs (m/z: 695) following LC-MS analysis of TCA precipitated 5’-IAF-labelled mycelial extracts from ATCC26933, Δ*egtA*^26933^, AfS77 and Δ*egtA*^AfS77^, in addition to a GSH standard. According to these data, total cellular GSH levels in ATCC26933 are lower compared to the other strains. (b) **Free** GSH and GSSG pools in ATCC26933, Δ*egtA*^26933^, *egtA*^C26933^, AfS77, Δ*egtA*^AfS77^ and *egtA*^CAfS77^.


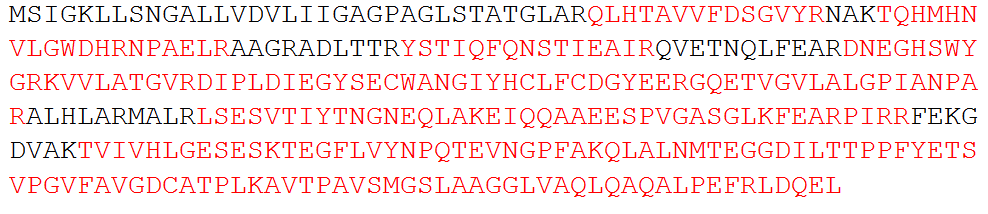


**Figure S7:** The amino acid sequence for GliT, with detected peptides highlighted in red (19 peptides, 77.2 % coverage). GliT showed significantly increased abundance (1.142 Log_2_-fold increase, P = 0.043) in Δ*egtA*^26933^ exposed to 5 µg/ml gliotoxin for 3 h compared to Δ*egtA*^26933^ with equivalent methanol exposure.

**A.**

**B.**

**Figure S8:** Northern analysis of **A.** *egtA*, **B.** *cat2* and **C.** *sidG* expression (in AMM cultures containing 20 mM L-glutamine as nitrogen source), in the presence and absence of 1 mM H_2_O_2_ & + FeSO_4_ in *A. fumigatus* AfS77 (wt), Δ*egtA (*Δ*e)*, Δ*yap1 (*Δ*y)*, Δ*egtA*::Δ*yap1(*Δ*e*Δ*y)*. Upper panels in each of **A**., **B**. and **C**. show RNA loading controls.

**C.**
